# Acute effects of psilocybin on attention and executive functioning in healthy volunteers: a systematic review and multilevel meta-analysis

**DOI:** 10.1007/s00213-024-06742-2

**Published:** 2025-01-23

**Authors:** P. Yousefi, Morten P. Lietz, F. J. O’Higgins, R. C. A. Rippe, G. Hasler, M. van Elk, S. Enriquez-Geppert

**Affiliations:** 1 Cognitive Psychology Unit, Institute of Psychology, Leiden University, Leiden, the Netherlands; 2 Molecular Psychiatry Lab, Faculty of Science and Medicine, University of Freiburg, Villars-sur-Glâne, Switzerland; 3 Department of Clinical and Developmental Neuropsychology, University of Groningen, Groningen, the Netherlands; 4 Royal College of Surgeons in Ireland, Dublin, Ireland; 5 Institute of Education and Child Studies, Leiden University, Leiden, the Netherlands; 6 Freiburg Mental Health Network, Villars-sur-Glâne, Switzerland; 7 Lake Lucerne Institute, Vitznau, Switzerland; 8 Department of Biomedical Sciences of Cells & Systems, University Medical Center Groningen, Groningen, the Netherlands; 9 Psychedelic Treatment and Mechanisms Group, University Centre of Psychiatry, Groningen, the Netherlands

**Keywords:** Psilocybin, Executive function, Attention, Cognitive performance, Reaction time, Accuracy, Meta-analysis, Working memory, Psychedelics, Acute effects

## Abstract

**Rationale:**

Psilocybin shows promise for treating neuropsychiatric disorders. However, insight into its acute effects on cognition is lacking. Given the significant role of executive functions in daily life and treatment efficacy, it is crucial to evaluate how psilocybin influences these cognitive domains.

**Objectives:**

This meta-analysis aims to quantify the acute effects of psilocybin on executive functions and attention, while examining how dosage, timing of administration, cognitive domain, and task characteristics moderate these effects.

**Methods:**

A systematic review and multilevel meta-analysis were conducted on empirical studies assessing psilocybin’s acute effects on working memory, conflict monitoring, response inhibition, cognitive flexibility, and attention. Effect sizes for reaction time (RT) and accuracy (ACC) were calculated, exploring the effects of timing (on-peak defined as 90–180 min post-administration), dosage, cognitive function categories, and task sensitivity to executive functions as potential moderators.

**Results:**

Thirteen studies (42 effect sizes) were included. In the acute phase, psilocybin increased RTs (Hedges' g = 1.13, 95% CI [0.57, 1.7]) and did not affect ACC (Hedges' g = -0.45, 95% CI [-0.93, 0.034]). Effects on RT were dose dependent. Significant between-study heterogeneity was found for both RT and ACC. Task sensitivity to executive functions moderated RT effects. Publication bias was evident, but the overall effect remained significant after adjustment for this.

**Conclusions:**

Our meta-analysis shows that psilocybin impairs executive functions and results in a slowing down of RT. We discuss potential neurochemical mechanisms underlying the observed effects as well as implications for the safe use of psilocybin in clinical and experimental contexts.

**Supplementary Information:**

The online version contains supplementary material available at 10.1007/s00213-024-06742-2.

Psilocybin has gained increasing interest in recent years due to its potential therapeutic effects on various neuropsychiatric disorders, including depression (Carhart-Harris et al. 2021; Li et al. 2022; McCartney et al. 2022; Więckiewicz et al. 2021), anxiety (Griffiths et al. 2016; Ross et al. 2016) and substance use disorder (Bogenschutz et al. 2022; Johnson et al. 2017). While the effects of psilocybin on emotions and psychological functioning are widely studied (Barrett et al. 2020a, b; Basedow et al. 2021; Irizarry et al. 2022; Nutt and Carhart-Harris 2021), research is increasingly focusing on its effects on cognition (Bonnieux et al. 2023; Sayalı and Barrett 2023). Insight in this will help to understand the mechanisms of action of therapeutic approaches using psychedelics, to assess safety and the potential of cognitive enhancement.

Cognitive impairments, in particular in the domain of executive functioning, are commonly observed across various forms of psychopathology, underscoring their transdiagnostic significance (Abramovitch et al. 2021; Snyder et al. 2015). Executive functions are top-down mental processes that coordinate other, lower-level cognitive abilities (e.g., attention) to enable goal-oriented actions and flexibly adjust behavior in novel and challenging environments (Friedman and Miyake 2017). Given the role of executive dysfunctions in various psychopathologies, it is crucial to evaluate how psychedelics may influence these cognitive functions over different time points and understand how moderating factors such as dose and time point of measurement play a role, to elucidate the potentially harmful or beneficial effects of psilocybin on cognition, thus furthering clinical applicability (Bălăeţ 2022).

Given the therapeutic potential of psilocybin for various neuropsychiatric disorders and the transdiagnostic significance of executive function impairments, understanding psilocybin’s effects on cognition is crucial. However, psychedelic research and specifically research on the effects of psychedelics on cognition, faces significant methodological challenges, including variability in placebo groups, potential expectancy effects, dosages, and administration protocols (Hendy 2018; Van Elk and Fried 2023). The use of different cognitive assessment measures across studies further complicates result interpretation. Additionally, individual factors such as “set and setting” can influence cognitive outcomes (Studerus et al. 2012; Hartogsohn 2017). These challenges, combined with the need to elucidate potentially harmful or beneficial effects of psilocybin on cognition for clinical applicability (Bălăeţ 2022), underscore the necessity for a comprehensive and critical review of psilocybin’s effects on cognition and that is the aim of the current study.

## Effects of psilocybin on executive functions and attention

A prominent data-driven model of executive functions (Miyake et al. 2000) identifies basic subcomponents: working memory updating, response inhibition, and shifting (cognitive flexibility). Neuroscientific findings further distinguish conflict monitoring from response inhibition as a distinct and vital aspect of executive function, (e.g. Enriquez-Geppert et al. (2010). These subcomponents support planning and problem-solving abilities (Miyake et al. 2000). Although most current studies have not focused on cognitive processes as a primary outcome, they have nevertheless made assessments of the effect of psilocybin on cognitive processes, such as attention (Cavanna et al. 2022), working memory (Barrett et al. 2018), and inhibition (Doss et al. 2021; Kometer et al. 2012; Marschall et al. 2021), using computerized or pen-and-paper-based cognitive tasks.

However, there are methodological challenges in traditional clinical measures of executive functions, known as the task impurity problem (Miyake and Friedman 2012). Many tasks designed to assess specific executive functions are inevitably influenced by other cognitive processes. For instance, Luciana and colleagues (2009) found that inattention is negatively correlated with Tower of London task performance, highlighting how attention can impact tasks meant to measure planning and problem-solving. Additionally, motor abilities can play a significant role in executive function tasks. Van Den Heuvel et al. (2003) demonstrated that Tower of London task performance was associated with activation not only in the expected dorsolateral prefrontal cortex but also in motor-related areas such as the striatum, premotor cortex, and supplementary motor area. This complexity of measuring executive functions becomes especially relevant when studying the effects of psychoactive substances like psilocybin, which may have broad impacts across multiple cognitive domains. Therefore, when interpreting the results of executive function tasks under the acute influence of psilocybin, it’s crucial to consider that observed changes in performance might reflect alterations in attention, motor function, or other basic cognitive processes, rather than, or in addition to, changes in the specific executive function being targeted.

This systematic review and meta-analysis evaluates the effects of psilocybin on cognition, focusing on executive functions (Miyake and Friedman’s CC). Using advanced meta-analysis methods, we aim to determine psilocybin’s acute pooled effects on RT and ACC across cognitive tasks measuring executive functions. We will also examine dose, measurement timing, cognitive subcomponents, and task measurement sensitivity as potential moderators of these effects. This is the first meta-analysis to comprehensively assess psilocybin’s impact on cognitive performance.

## Methods

### Literature search

A systematic review was conducted by searching multiple electronic databases, including PubMed, PsychInfo, Web of Science, and Cochrane) to identify empirical articles on psilocybin and executive functions using the key search terms *cognition* or cognitive function* or executive function* or *cognitive control* or *inhibition* or *memory updating* or *conflict monitoring* or *task switching* or *set-shifting*, combined with one of the following terms: psychedelic* or hallucinogen* or psiloc* or *psychotomimetic* or entheog* or *shrooms*. We searched for articles during the months of July and August 2022. The search was updated once in July 2023.

To meet the inclusion criteria, articles reporting on original studies had to meet the following requirements: They had to measure at least one of the following cognitive domains under the influence of psilocybin: (a) working memory (updating) (b) conflict monitoring c) response inhibition (d) cognitive flexibility or (e) attention.

Exclusion criteria encompassed studies that (1) were not written in English, (2) did not involve psilocybin administration, (3) used an inappropriate study design that did not fulfill our objective criteria (animal models, or lack of executive function measures), (4) were of an incorrect publication type (background article, reviews, dissertation) or (5) were inaccessible.

The software Rayyan (Ouzzani et al. 2016) was used for screening abstracts, and the detection of duplicates. Three authors (PY, ML, FO) were responsible for independently screening abstracts of each study. For the exclusion of a study, the assessment of only one author was sufficient. However, for the inclusion of the study, at least two authors had to include the study. Disagreements were addressed by the decision of the third author.

### Data extraction

To systematically collect data, four authors (PY, ML, FOH, SEG) investigated the full-text articles of the selected studies from September to November 2023. For data extraction, multiple outcome domains were targeted: cognitive function, specific cognitive measures, sample size, dependent variables for each cognitive measure, dosage, measurement time points, and either pre-calculated effect sizes or the raw data required to calculate them. For labeling purposes, the dosages of the included studies were categorized according to the following categories: 1–5 mg micro, 6–19 mg: low, 20–30 mg: medium, > 30 mg: high (all per 70 kg). In case of missing data for calculating effect sizes, the corresponding author of the respective study was contacted via email.

For the meta-analytical procedures, the focus was on specific cognitive outcome measures of RTs and ACC. Effect measures for outcomes were given as means and median differences, which were converted into standardized values and as the basis for the calculation of Cohen’s d for each study. The scripts for the calculation of the effect sizes for each study and the extracted data used for these calculations can be found in the supplementary material. For the extraction of data from graphical representations in individual studies, the software WebPlotDigitizer (Rohatgi 2022) was utilized (PY and FOH), and results were double checked by a second rater (ML).

### Risk of bias assessment

Two independent authors (FOH and ML) employed the Cochrane Risk of Bias 2 assessment tool (Higgins et al. 2023) to systematically evaluate the risk of bias in trials included in this study. They evaluated each trial based on five specific domains: randomization, deviations from the intended intervention, missing data, outcome measurement, and the selection of the reported outcome. Each domain was categorized as having a low, some concern, or high risk of bias. Studies with a high risk of bias in any single domain were noted as having high risk of bias in the overall evaluation, while those with some risk of bias in one or more domains were classified as having some concerns overall in terms of risk of bias. The assessments of both raters were combined and visualized using the Risk-of-Bias Visualization software (robvis) developed by McGuinness and Higgins (2020). Following the evaluations, an interrater reliability analysis was conducted to determine the consistency of the presence and level of bias identified.

To assess the potential risk of publication bias, funnel plot analysis complemented by Kendall’s rank correlation test were used. Additionally, Rosenthal’s, Rosenberg’s, and Orwin’s fail-safe numbers were calculated to determine the number of unpublished studies required to negate the observed effect size.

### Meta analysis

A multilevel meta-analysis was chosen to accommodate the complex structure of the data, specifically the fact that multiple effect sizes were extracted from single studies. The analysis was initiated with a multilevel random-effects model, allowing for the assessment of within-study and between-study variances. Hedges’ g was selected as the effect size measure. The Bayesian information criterion (BIC) and the Akaike information criterion (AIC) were employed for model comparisons.

Heterogeneity among study results was quantified using the I² statistic and the Q statistic. The robustness of the findings was tested by comparing results from the multilevel model with those from a simpler non-nested random-effects model. Further analysis involved exploring potential moderators such as dosage, cognitive functional categories, and timing relative to peak psilocybin.

**Table 1:** Cognitive tasks and their evaluated sensitivity to executive functions or attention

	Nr of extracted EFs	Task Sensitivity score
Letter-N-Back	3	1
Emotional Stroop	3	3
Digit Symbol Substitution Test	10	3
Spatial Span Test	5	2
Attentional Object Tracking	1	1
Stroop	4	1 & 2*
Go/NoGo	1	1
Emotional Go/NoGo	4	3 & 1**
Attentional Blink	2	1
Trail Making Test	1	2
Covert Orienting of Attention Task	1	1
Frankfurt Attention Inventory	5	2
Psychomotor Vigilance Task	1	2
Spatial Memory Test	1	2
Tower of London	1	3
Delayed Response Task	1	3

* 2 for Study_ID = 8; ** 1 for ES_ID = 31; Task Sensitivity score (1 = high, 2 = medium, 3 = low)

Furthermore, we categorized each effect size based on its sensitivity to measure executive functioning or attention: 1 = high sensitivity (i.e. mean difference scores between the task condition assessing the function of interest and the baseline condition, e.g., in a Stroop task, RT of incongruent condition subtracted from congruent condition), 2 = medium sensitivity (i.e. mean score of task condition assessing the function of interest, e.g., in Stroop task, incongruent condition RTs ), and 3 = low sensitivity (i.e., mean score across all task conditions, e.g.in Stroop task, main effect of drug averaged across incongruent and congruent conditions). This variation may impact the validity of the results, as it complicates the precision by which aspects of executive functioning are actually being measured. An overview of each task in our dataset and the corresponding sensitivity value, judged based on the data available to us, is presented in Table 1.

Statistical procedures were conducted using R (v4.3.2; R Core Team, 2020), RStudio (Rstudio Team 2020), the main multilevel analysis with the metafor package (Viechtbauer 2010). For further details on other used packages referrer to the supplementary code.

Following the approach suggested by Viechtbauer and Cheung (2010), a study was considered an outlier if its confidence interval did not overlap with the confidence interval of the pooled effect (Viechtbauer and Cheung 2010). To identify influential cases, we employed three diagnostic measures: Cook’s distance, hat values (leverage), and DFBETAS. Cook’s distance assesses the influence of each study on the overall meta-analysis results, with values greater than 4/(n-2) considered potential outliers, where n is the number of studies. Hat values measure the influence of each study on the fitted values, and studies with hat values exceeding twice the mean hat value were deemed potential outliers. DFBETAS evaluates the influence of each study on the estimated coefficients, and studies with absolute DFBETAS values larger than 2/sqrt(n) were considered potential outliers.

## Results

We first discuss study selection and present a narrative review of psilocybin’s effects on various cognitive domains. We then report the results of our meta-analysis, examining psilocybin’s acute effects on reaction times and accuracy across different cognitive tasks, including moderation analyses for factors such as dosage, timing, and task sensitivity. Finally, we assess the publication bias.

**Table 2:** Overview Included studies and study IDs

Study	Study ID	Nr of tasks	number of extracted effect sizes		
			Total	ACC	RT
Barrett et al (2018)	1	3	15	9	6
Carter et al (2005)	2	2	2	2	0
Cavanna et al (2022)	3	3	6	3	3
Doss et al (2021)	4	0	0	0	0
Gouzoulis-Mayfrank et al. (2002)	5	1	1	0	1
Hasler et al (2004)	6	1	4	4	0
Kometer et al (2012)	7	1	1	1	0
Quednow et al (2011)	8	1	2	1	1
Vollenweider et al (2007)	9	1	1	1	0
Wittmann et al (2006)	10	1	4	4	0
Marschall et al (2021)	11	1	1	1	0
Mallaroni et al (2023)	12	4	4	1	3
Vollenweider et al. (1998)	13	1	1	0	1
Total			42	27	15

### Study selection

**Fig. 1:**
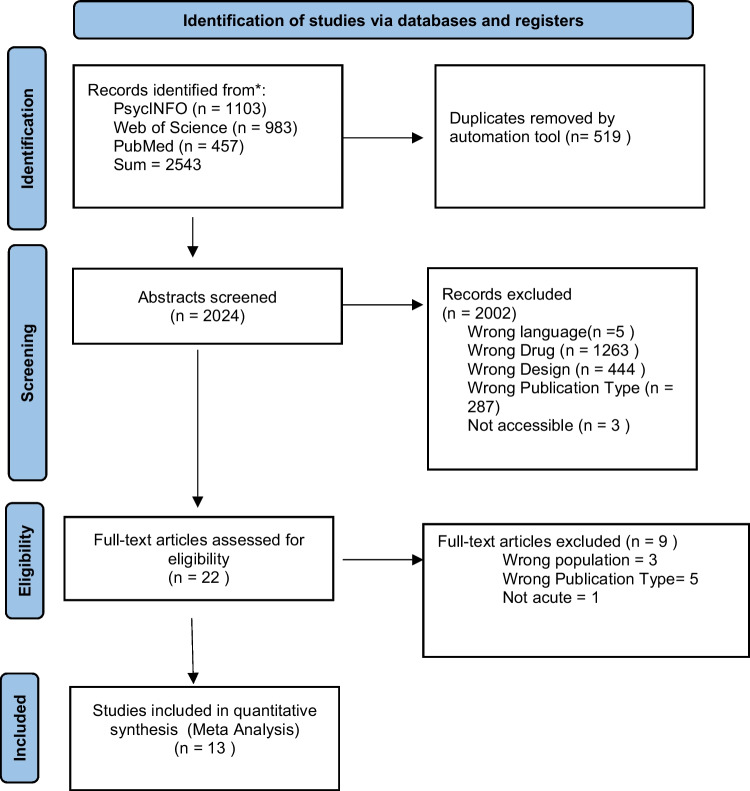
PRISMA flow diagram illustrating the study selection process (Page et al. 2021).

The search yielded a total of 2543 articles, which were screened by title (Fig. 1). Articles that did not meet the inclusion criteria were excluded at this stage. The remaining articles were uploaded into Rayaan (Ouzzani et al. 2016), duplicates were removed, and the abstracts (and full-text where applicable) were screened. The screening process was conducted by four independent authors. Eventually, a total of 13 studies were suited for the present systematic review (Table 2). One study (ID = 4) initially included in the literature review was post-hoc excluded from the subsequent meta-analysis, as this study was the only study where the measurement time point was days after substance administration, unlike the other studies with measurement time points ranged from 60 to 360 min.

### Literature review

The following section describes the effects of psilocybin on executive functions and attention and is organized according to the four components of executive functions: working memory (updating), conflict monitoring, inhibition, cognitive flexibility, and attention. The included studies used a variety of tasks to assess these different aspects of executive functions and attention. An overview of these tasks and their outcome measures is presented in Table 3.

**Table 3:** Overview of cognitive measures used in the included studies

Domain		Task	Study IDs	Description	Variables
Specific Executive Function	**Working memory (updating)**	N-back	(1)	The N-Back task is a measure of working memory (updating). Participants view a sequence of letters and must indicate whether the current letter matches the one presented "n" steps back.	(1): Discriminately rate (hit rate [HR] - false alarm rate [FAR]),Response bias (FAR/[1-ACC]), RT
		Spatial Memory Task	(12)	The Spatial Memory Test (SMT) is used mainly in psychopharmacological drug research to assess visuospatial memory and reasoning. It has two phases: immediate and delayed recall. In the immediate recall, participants view six sets of ten black-and-white images on a computer screen, each for 2 seconds with a 1-second gap in between. Subsequently, they must identify the original location of each image as it reappears on the screen. After a 30-minute interval, the delayed recall phase requires participants to recall the locations of the pictures.	(12): Mean ACC, Mean RT
		Delayed Response Task		The delayed-response task is a cognitive test that evaluates working memory, focusing particularly on spatial working memory. In this task, participants view a stimulus briefly displayed in a specific location on a touch screen. After the stimulus disappears, a numerical distraction task fills a ten-second delay period to prevent the rehearsal of the stimulus location. Subsequently, participants must accurately recall and indicate where the stimulus was located by touching the corresponding area on the screen. This task measures the ability to retain and manipulate visuospatial information over short periods without external cues and is commonly used to explore the functions of the dorsolateral prefrontal cortex, a key area for memory and executive functions.	(13): RT
		Spatial Span Test	(10) (2)	The spatial span test is a measure of visual-spatial working memory performance. Nine white squares are randomly displayed on a black background on a touch screen. On each trial, some of the squares are highlighted at a specific interstimulus interval. Participants indicate the order of the highlighted squares. The trial starts with two squares and increases up to nine. The test ends after three wrong answers or reaching a maximum of 9 squares (Max. score). The number of squares correctly reproduced is the span-length.	(2): Span-length,# of errors(10): Span-length
	**Conflict Monitoring**	Stroop task	(4) (3) (8)	The Stroop task is a measure of conflict monitoring. Typically a colour word is presented in a specific colour. Participants have to indicate the ink colour of the colour words, which can either be congruent (e.g., the word "green" in green colour) or incongruent (e.g., the word "green" in blue colour). The incongruent condition usually results in longer RTs and more errors, illustrating the cognitive conflict between word reading and colour naming ("Stroop effect").	(3): RT, ACC (4): Hit Rate, median RT, Signal detection (discrimination d')(8): RT, # errors, Interference, facilitation, Stroop effect
		Emotional Stroop task	(1)	The emotional Stroop task is a measure of emotional conflict monitoring. Participants are presented with words that are emotionally salient and neutral words. Participants indicate the colour in which the words are presented.	(1) RT, ACC
	**Response Inhibition**	Go/NoGo	(3)	The Go/NoGo task is a measure of response inhibition. Participants are required to respond (Go) or withhold their response to a specific stimuli (NoGo) , allowing for the measurement of the ability to inhibit prepotent responses.	(3): ACC
		emotional Go/NoGo	(7) (11)	The emotional Go/NoGo task is a measure of emotional inhibition. Participants see affective stimuli (e.g., positive, negative, or neutral words) and respond only to a specific emotional category (e.g., positive) while withholding a response to another (e.g., negative or neutral).	(7): RT, ACC, Sequential effects(11): RT, ACC
	**Cognitive Flexibility**	Digit-Symbol Substitution Task	(1) (12)	The Digit-Symbol Substitution Task (DSST) evaluates cognitive flexibility through rapid task-switching and adaptive strategy use. Participants must flexibly apply symbol-digit pairings, quickly shifting between different associations as they fill in a series of boxes. This constant adaptation of mental sets demonstrates cognitive flexibility in real-time problem-solving.	(1): Total # of attempted trials, ACC (% correct)(12): Total #Reponses,Total #Correct, Mean RT
		Penn Conditional Exclusion Test	(4)	The Penn Conditional Exclusion Test (PCET) directly assesses cognitive flexibility, mirroring the Wisconsin Card Sorting Test. By requiring participants to identify common characteristics among item sets, it measures their ability to flexibly shift mental strategies and adapt to changing rules - key components of cognitive flexibility.	4): Preservation errors, Median RT, Correct, incorrect, total responses
		Trail Making Test	(3)	The Trail Making Test, particularly Part B, is a prime measure of cognitive flexibility. While Part A involves simple sequencing, Part B demands flexible alternation between numbers and letters. This task switching directly taps into cognitive flexibility, requiring participants to mentally shift between two sequences, adapting their approach throughout the test.	(3): Time to finish the task, Number of errors
		Tower of London	(12)	The Tower of London (TOL) task evaluates cognitive flexibility within the context of planning and problem-solving. Participants must flexibly generate and modify strategies to rearrange beads with minimal moves. This constant adaptation of plans and approaches as the task progresses demonstrates cognitive flexibility in complex problem-solving scenarios, even in individuals with severe cognitive impairments.	(12): Total correct trials, RT
**Attention**		Frankfurt Attention Inventory	(6) (9)	Frankfurt Attention Inventory (FAIR) is a paper-pencil measure of sustained and directed attention. Participants identify target stimuli within distractor items within a limited time by spiking at each target and drawing a continuous line below the distractors. The test yields three main scores: Performance score (P): Measures overall effectiveness in completing the task. Quality score (Q): Reflects ACC, calculated as the ratio of correct decisions to total decisions made. Continuity score (C): Assesses the consistency of attention throughout the task duration.	(6)(9): Performance score (P) / Marker Value, Quality score (Q), Continuity score (C)
		Attentional blink task	(3)	The attentional blink (AB) task is a measure for allocating attention over time. Participants view a rapid stream of letters with two-digit targets (T1 and T2) inserted. The aim is to identify both digits, and performance is evaluated based on correct detection rates. When T2 appears with a short stimulus onset interval, T2 is often not detected. The task incorporates varying time lags between the targets to assess the duration of the attentional "blink".	(3): Visibility rate of targets (T1,T2)
		Covert orienting of attention task	(5)	The Covert orienting of attention task (COVAT) assesses attentional control by requiring participants to quickly respond to visual stimuli appearing in different locations on a screen. Cues indicate where the stimulus may appear, but they may be misleading.	(5): RT, Validity effect (difference between invalid and valid RTs)
		Multiple Objects Tracking	(2)	Multiple Objects Tracking (MOT) is a measure of divided visual attention to moving visual stimuli. During the task, participants keep their gaze on a fixation cross and look at a small set of identical stimuli of targets and distractors that start moving randomly on a display. Participants observe these moving objects among distractors and identify them post-movement selecting the objects they believe were the original targets.	(2): The mean % of correct responses, Mean # of successfully tracked targets, Discriminability index

#### Acute psilocybin effects on working memory (updating)

Barrett et al. (2018) found that psilocybin acutely and selectively affects working memory in a dose-dependent manner. Using the Letter-N-Back task 180 min post-psilocybin administration, they observed significantly lowered discriminability, increased response bias, and prolonged response time during the 2-back condition compared to placebo. These effects on RT were more pronounced at higher doses (20–30 mg/70kg; d(20 mg) = 1.36, large effect; d(30 mg) = 1.89, large effect) compared to lower doses (10 mg/70kg; d(10 mg) = 1.25, large effect).

In contrast, Carter et al. (2005) used the Spatial Span Test and reported that psilocybin did not significantly affect spatial working memory span or errors acutely at a dose of 15 mg, even though the psilocybin group did make more mistakes in their sample (d= −0.62, medium effect). This suggests a potential dissociation between the effects of psilocybin on different aspects of working memory functions (updating vs. span) or tasks.

Wittmann et al. (2006) found that psilocybin acutely reduced spatial span length at 100 min post-psilocybin administration of a low dose (17.5 mg/kg; d = −0.31, small effect) but not at a lower dose (8.05 mg/70kg; d(100 min)= −0.04, no effect; d(360 min)= −0.12, no effect) or at a later time point of 360 min post-psilocybin administration (d = 0.02, no effect).

Mallaroni et al. (2023) compared the acute effects of psilocybin and 2 C-B on different cognitive functions. Both substances impaired global cognitive function, including working memory, as measured by the reduction in correct responses (d = −1.34, large effect) in the psilocybin group during the Spatial Memory Task at 225 min post-psilocybin administration.

Vollenweider et al. (1998) investigated the role of serotonin receptors in psilocybin-induced working memory effects. They found that psilocybin prolonged RTs on a delayed response task at 80 min post-psilocybin administration (d = 1.75, large effect). These acute increases were prevented by pretreatment with serotonin-2 antagonists but not dopamine antagonists, suggesting that the effects are primarily mediated by serotonin-2 A receptor activation.

To summarize, studies on psilocybin’s acute effects on working memory show mixed results. While some report dose-dependent impairments in updating and global cognitive function, others find no significant effects on spatial working memory span. The impact appears to vary based on task type, dosage, and assessment timing. Evidence suggests these effects are primarily mediated by serotonin-2 A receptor activation.

#### Acute psilocybin effects on conflict monitoring

Several studies have investigated the effects of psilocybin on conflict monitoring using various cognitive tasks. Barrett et al. (2018) found that psilocybin induced dose-dependent effects in conflict monitoring as assessed by the emotional Stroop task. RTs increased significantly with increasing doses of psilocybin across the incongruent and congruent conditions (10, 20, and 30 mg/70kg; d = 1.44, 2.1, 2.5; all large effect sizes) compared to placebo at 240 min post-psilocybin administration. However, the study did not find a significant effect on ACC.

Cavanna et al. (2022) investigated the effects of psilocybin microdosing (0.795 mg/70kg) using the Stroop. At 180.

minutes post-psilocybin administration, participants exhibited longer RTs (d = 0.51; medium effect) and lower ACC (incongruent - congruent condition; d = −0.11; small effect) in the Stroop task under psilocybin compared to an inactive placebo (edible mushroom). These findings suggest that even at microdoses, psilocybin may slightly slowdown conflict monitoring.

Quednow et al. (2011) examined the effects of a low dose of psilocybin (18.5 mg/70kg) using the Stroop task. At 85 min post-psilocybin administration, psilocybin increased RTs (d = 1.03; large effect) and decreased ACC (d = −0.85; large effect). The authors attributed these effects to the stimulation of serotonin-2 A receptors by psilocybin, as pretreatment with the 5-HT2A/2 C receptor antagonist ketanserin attenuated these effects.

Doss et al. (2021) explored the long-term effects of psilocybin treatment on conflict monitoring in patients diagnosed with Major Depressive Disorder (MDD). This open-label clinical trial involved 24 participants, administering either a medium dose of 20 mg/70 kg or a high dose of 30 mg/70 kg of psilocybin. Assessments were made at multiple time points: eight weeks before, at baseline, and one and four weeks after the treatment. Psilocybin showed no significant effect on RT or ACC in the Stroop task.

*To summarize*, the four studies reviewed consistently demonstrate that psilocybin affects conflict monitoring in a dose-dependent manner. Higher doses of psilocybin lead to more pronounced increases in RTs and decreases in ACC on tasks involving conflict resolution. These effects are evident even at microdoses.

#### Acute psilocybin effects on response inhibition

Cavanna et al. (2022) investigated the effects of psilocybin microdosing (0.795 mg/70kg) on inhibition using the Go/No-Go task. At 150 min, the study found no significant differences in response ACC between the psilocybin and placebo conditions. However, there was a slight decrease in ACC in their sample (NoGo-Go condition; d =−0.01; very small effect).

Kometer et al. (2012) examined the effects of a low dose of psilocybin (15.05 mg/70kg) on inhibiting emotional stimuli using the emotional Go/No-Go task. At 120 min post-psilocybin administration, psilocybin decreased ACC (d = −2.16; large effect) and increased RTs (d = 1.56; large effect) compared to placebo. The increase in RT was modulated by the valence of the words used in the task. Specifically, the psilocybin group exhibited longer RTs for negative words compared to positive suggesting an increased effect on negative cognitive control processing under the influence of psilocybin.

Marschall et al. (2021) also investigated the effects of psilocybin microdosing (1.5 mg/70kg) on inhibition using the emotional Go/No-Go task. At 90 min post-psilocybin administration, the study found no significant effect on RTs or ACC (d = =−0.03; small effect) in the No-Go trials between the psilocybin and placebo (edible mushroom) conditions.

*To summarize*: The studies indicate that while low doses of psilocybin significantly impair ACC and increase RTs, particularly for emotional stimuli, microdoses generally show negligible effects on these measures.

#### Acute psilocybin effects on attention

Several studies have investigated the effects of psilocybin on various aspects of attention. Carter et al. (2005) found that psilocybin (15.05 mg/70kg) significantly reduced the ACC of attentional tracking at 120 min post-psilocybin administration (effect size = −1.305). This might indicate a reduction in the ability to accurately track multiple objects.

Cavanna et al. (2022) investigated the effects of psilocybin microdosing (0.795 mg/70kg) on attention using the attentional blink task at 180 min post-psilocybin administration. Our analysis on the raw data revealed that RT was reduced (d= −0.04; no effect) and ACC was increased (d = 0.3; small effect), suggesting that psilocybin microdosing enhances ACC and slightly reduces RT in attentional tasks (see supplementary material for detailed methodology).

Gouzoulis-Mayfrank et al. (2002) found that psilocybin (14 mg/70kg) significantly prolonged RTs in the Covert Orienting of Attention Task compared to placebo at 85 min post-psilocybin administration (d = 0.47; small effect). In particular, subjects had difficulty disengaging attention from the cued location and reorienting it to the target in the opposite visual field, especially for targets in the right visual field. The authors suggested a potential lateralized psilocybin effect in the visuospatial attentional network, particularly affecting the right hemisphere.

In the study by Hasler et al. (2004), psilocybin affected the Quality Value (QV) scores in a dose-dependent manner. The QV scores in the Frankfurt Attention Inventory reflect the ACC of attentively made decisions. A microdose (3.15 mg/70kg) and low dose (8.05 mg/70kg) of psilocybin slightly increased QV scores (d = 0.4 and d = 0.62, respectively). However, the medium dose (15.05 mg/70kg) and high dose (22.05 mg/70kg) decreased QV scores (with d= −0.25; small effect; and − 0.58; medium effect, respectively), indicating a reduction in ACC at higher doses.

Vollenweider et al. (2007) also found that psilocybin dose-dependently effects on sustained attention as measured by the FAIR at 105 min post-psilocybin administration. The Performance Value scores were significantly reduced by low (8.05 mg/70kg d = −1.03 and 15.05 mg/70kg d = −1.27; large effect), and medium (22.05 mg/70kg; d =−1.17; large effect) doses of psilocybin. The Quality Value score, reflecting ACC, was also significantly reduced by the medium dose (d = −0.95; large effect).

Mallaroni et al. (2023) reported that psilocybin (15 mg/70kg) selectively increased RTs on the psychomotor vigilance task compared to placebo at 166 min post-psilocybin administration (effect size = 0.81; large effect), although it did not significantly impair overall performance or ACC on this task of sustained attention.

*To summarize*: While some studies suggest that psilocybin impairs attentional processes, such as attentional tracking (Carter et al. 2005), reorienting attention (Gouzoulis-Mayfrank et al. 2002), and sustained attention (Hasler et al. 2004; Vollenweider et al. 2007), one indicated a potential enhancement in specific aspects of attention, particularly at microdoses (Cavanna et al. 2022). The effects of psilocybin on attention appear to be more pronounced at higher doses, with medium and high doses leading to significant reductions in ACC and performance on attentional tasks.

#### Acute psilocybin effects on cognitive flexibility

Three studies have investigated the effects of psilocybin on cognitive flexibility using the Digit Symbol Substitution Task (DSST), the Tower of London (TOL), and the Trail Making Test (TMT).

Barrett et al. (2018) found that psilocybin caused a dose-dependent decrease in the number of trials which were attempted by the participants in the DSST, indicating a reduction in processing speed. This effect was observed at one, two, and three hours post-psilocybin administration for low (10 mg/70kg, d =−0.67; medium effect), medium (20 mg/70kg, d= −1.47; large effect), and high (30 mg/70kg, d= −2.32; large effect) doses. Interestingly, while the number of attempted trials was reduced, the ACC of responses (i.e., the proportion of correct trials out of all attempted trials) was slightly increased (low dose: d = 0.17; small effect, medium dose: d = 0.08; small effect, high dose: d = 0.3; medium effect). This suggests that although psilocybin slows psychomotor speed, it may allow for compensatory strategies to maintain or even improve ACC, indicating a complex interaction between dosage and cognitive processes.

Mallaroni et al. (2023) showed that psilocybin (15 mg/70kg) led to lower performance on the digit symbol substitution task compared to placebo (edible mushroom). At 172 min post-administration, psilocybin increased RTs (d = 1.75; large effect) without significantly affecting ACC. These findings align with those of Barrett et al. (2018), indicating that psilocybin selectively impairs processing speed while preserving ACC on the DSST. In addition to the DSST, Mallaroni et al. (2023) used the tower of London task to assess the effects of psilocybin on planning and problem-solving abilities. At 153 min post-psilocybin administration, psilocybin (15 mg/70kg) increased RTs (d = 1.8) compared to placebo, suggesting a reduction in planning efficiency. However, the ACC of task performance was not significantly affected. These results indicate that psilocybin slows down cognitive processes involved in planning and problem-solving.

Cavanna et al. (2022) investigated the effects of psilocybin microdosing (0.795 mg/70kg) using the Trail Making Test (TMT). For Part B of the TMT, which involves alternating between numbers and letters in sequence, participants took significantly longer to complete the task under the psilocybin condition compared to the placebo (d = 0.76; medium effect) at 60 min post-administration. This result suggests that even at microdoses, psilocybin can impair cognitive flexibility and task-switching abilities.

To summarize, the three reviewed studies consistently demonstrate that psilocybin impairs cognitive flexibility, particularly in processing speed and planning efficiency. These effects seem dose-dependent, with higher doses leading to more pronounced effects. Interestingly, while psilocybin slows down cognitive processing, it does not significantly compromise the ACC of task performance in the Digit Symbol Substitution and Tower of London tasks.

### Meta analytic results: acute effects of psilocybin on reaction time

**Fig. 2:**
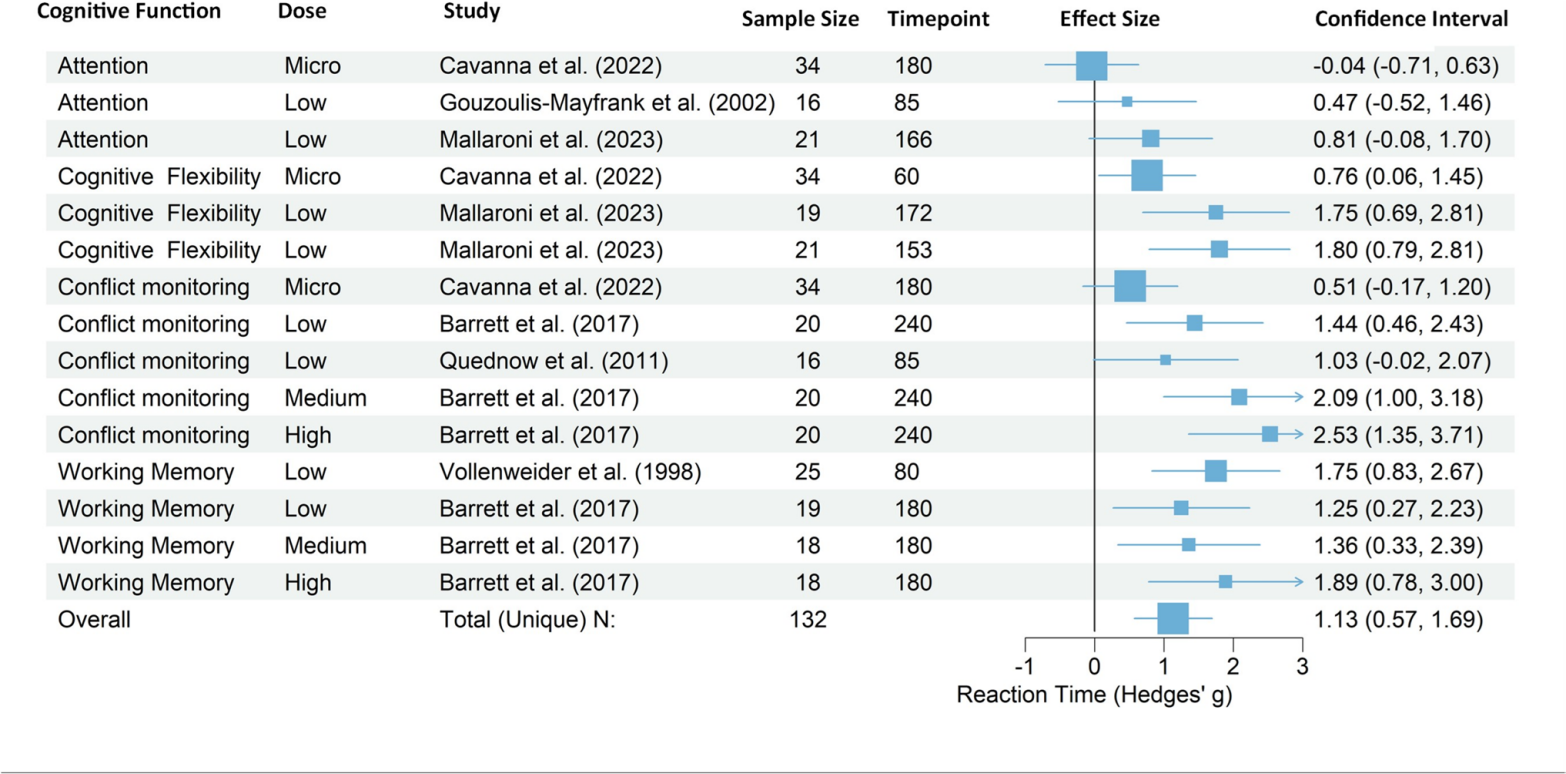
Acute effects of psilocybin on reaction time. Note: Forest plot of effect sizes (Hedges’ g) for psilocybin’s impact on RT. Results are sorted by cognitive domain, showing individual study effects and the overall pooled effect. Positive values indicate increased RT with psilocybin compared to placebo. The size of the squares indicates the relative weight of each study, with larger squares representing larger sample sizes. Measurement timepoint is displayed minutes. Error bars are truncated using arrows, in case of upper bounds being outside of figure bounds. There is an overall increase of RT

The acute effects of psilocybin on RT across different doses and studies are summarized in a forest plot (Fig. 2). There were no outliers identified for the RT dataset based on the criterion of non-overlapping confidence intervals of a single study with the pooled effect. One influential case (effect size id = 35) was found to have substantial leverage. Another case (effect size id = 7) was found to be an outlier because of very high standardized residuals (> 2). Removing these effect sizes and re-running the analysis yielded a slightly reduced but still significant overall hedge’s g of 1.20 (SE = 0.29, t = 4.16, df = 12, *p* = 0.0013, 95% CI [0.57, 1.83], and significant heterogeneity (I^2^ Total = 39%, *p* = 0.001), suggesting the robustness of the measured effect (see supplementary material for more details). These two cases were not excluded, as the heterogeneity without outliers was slightly higher than the heterogeneity in the model with outliers.

The dataset of RTs included 15 effect sizes (see Table 1) from six unique studies. A multilevel random effects meta-analysis with three levels, accounting for the nesting of multiple effect sizes within the same study, revealed an overall increase in RT under the influence of psilocybin (Hedges’ g = 1.13, SE = 0.26, t = 4.33, *p* = 0.0007, 95% CI [0.57, 1.7]).

The estimated variance components (the random-effects variances calculated for each level of our model) showed a between-study heterogeneity variance of σ^2^_Level3_ = 0.27 and a within-study variance of σ^2^_Level2_ = 0.015. Hereby σ^2^ represents the variance of the true effect sizes underlying the data. The total heterogeneity was moderate and significant (I^2^ Total = 36.77%, *p* = 0.0024). The precise amount of heterogeneity variance captured by each level was as follows: I^2^_Level3_= 34.88% of the total heterogeneity can be attributed to between-study differences, and I^2^_Level2_= 1.89% to within-studies differences. Overall, this indicates that there is between-study heterogeneity. Only a small fraction of the total variance can be explained by differences within studies.

The comparison of the full model with the reduced model using the likelihood ratio test revealed that the additional parameters in the full model improved model performance significantly (𝝌12 = 4.93, *p* = 0.0263). The Akaike Information Criterion (AIC) and Bayesian Information Criterion (BIC) were slightly lower for the full model, indicating that the nested model with more parameters provides a significantly better fit to the data than the reduced model. The Q statistic for heterogeneity was the same for both models, suggesting that the difference in model fit is not due to a change in how the models account for heterogeneity.

#### Influence of peak drug effects on reaction time

The peak window boundaries were defined to explore the main effect in the moderation analysis. This decision was based on the work of Holze et al. (2023), which documented that the time to maximal subjective effects of psilocybin, across different dosages (15, 25, and 30 mg), typically centered around two hours post-administration, with a reported range slightly extending from 1.7 to 2.4 h. To accommodate this range and ensure coverage of the peak subjective effects, we decided to conduct the moderation analysis using a 90–180 min interval.

We categorized studies and tasks as either falling within the defined peak boundary of 90–180 min (reference category) or outside this interval. Among the studies analyzed, eight effect sizes fell within the peak window, while seven were outside. The moderation analysis indicated that the use of this peak window as a moderator did not yield a statistically significant effect (QM (df = 1) = 2.19, *p* = 0.1387). This suggests that acute effects of psilocybin on RT during the peak were not different than before and after the peak.

#### Dosage-dependent effects of psilocybin on reaction time

Dosage was categorized into four levels: micro (the reference category), low, medium, and high. The analysis identified significant moderation by dosage (QM (df = 3) = 20.78, *p* = 0.0001), indicating that RTs varied significantly across different dosage levels. The intercept, representing the micro dosage level, approached significance, suggesting a potential increase in RTs at this minimal dosage level (estimate = 0.4, SE = 0.21, z = 1.88, *p* = 0.0589, 95% CI = −0.02 to 0.82). The effect sizes increased with each increasing dosage level: the low dosage already showed a significant increase in RTs (estimate = 0.87, SE = 0.28, z = 3.12, *p* = 0.0018, 95% CI = 0.32 to 1.42); the medium dosage continued this trend (estimate = 1.3, SE = 0.44, z = 2.92, *p* = 0.0035, 95% CI = 0.43 to 2.17); and the high dosage exhibited the largest increase (estimate = 1.79, SE = 0.47, z = 3.8, *p* = 0.0001, 95% CI = 0.87 to 2.72). These findings suggest a dose-response relationship where higher doses are associated with greater increases in RTs. The Test for Residual Heterogeneity indicated no significant residual heterogeneity (QE (df = 11) = 11.02, *p* = 0.4412), confirming that the variability among study outcomes is adequately captured by the dosage categories, affirming that the model appropriately accounts for differences across studies.

#### Impact of cognitive function and task sensitivity on reaction time

For the moderation analysis of cognitive function categories (Attention, Working Memory, Conflict Monitoring, Cognitive Flexibility; inhibition was missing in this subset) in the RTs dataset, the overall test for cognitive function as a moderator was not significant (QM (df = 3) = 5.7613, *p* = 0.1238), suggesting that variations in cognitive functions did not strongly influence the observed slowing of RT.

As described in our methods, we categorized each effect size based on its sensitivity to executive functioning or attention: Type 1 = pure (e.g., RT difference between incongruent and congruent conditions, reflecting a specific executive function process like conflict monitoring), Type 2 = specific executive function condition (e.g., performance on incongruent trials only), and Type 3 = executive and other cognitive functions (e.g., main effect of drug averaged across all task conditions). This categorization aimed to differentiate between tasks that isolate specific executive processes and those that involve multiple cognitive functions.

The moderation analysis of these sensitivity levels revealed significant differences (QM (df = 2) = 9.16, *p* = 0.0103). With Type 3 (executive and other cognitive functions) as the reference category, the model results indicated a robust baseline effect size (estimate = 1.69, SE = 0.27, *p* < 0.0001, CI = 1.15 to 2.23). This suggests that tasks involving multiple cognitive functions are most sensitive to the effects of psilocybin. In contrast, Type 1 (pure executive function measures) showed a significantly lesser effect (estimate = −0.92, SE = 0.34, *p* = 0.0072, CI = −1.59 to −0.25). This indicates that when tasks isolate specific executive processes, the effect of psilocybin is less pronounced. Type 2 (specific executive function conditions) also exhibited a reduced, small, effect compared to Type 3 (estimate = −0.68, SE = 0.39, *p* = 0.049, CI = −1.43 to −0.0025).

#### Evaluation of publication bias in reaction time studies

**Fig. 3:**
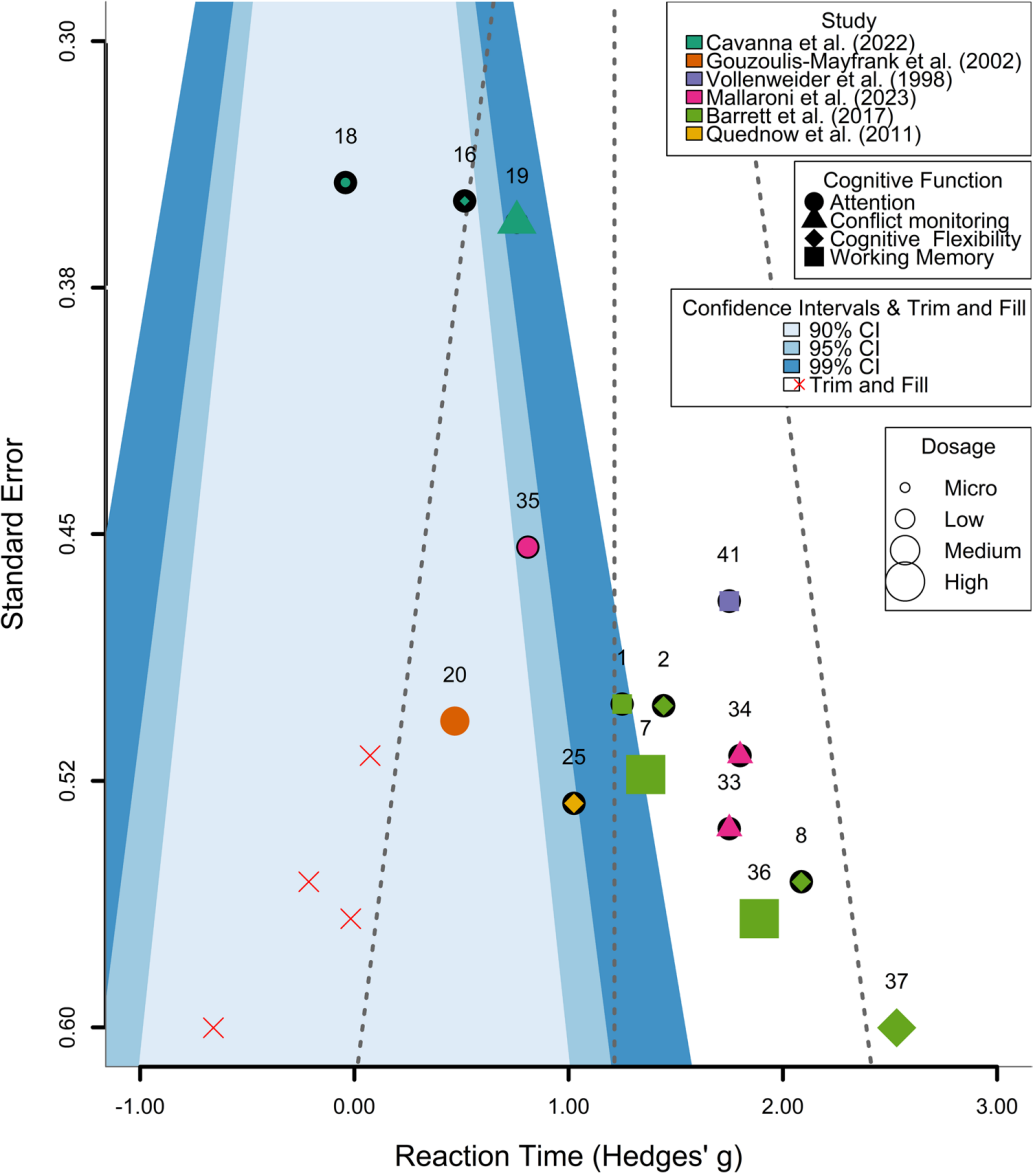
Publication bias assessment for psilocybin’s effects on reaction time. Note: Funnel plot of effect sizes for psilocybin’s impact on RT across studies. Points represent individual study outcomes, differentiated by cognitive function (shape), study (color), and dosage (size). Shaded areas indicate confidence intervals. Red crosses show trim-and-fill adjustments for potential publication bias. The plot suggests some asymmetry, with smaller studies showing more variable and stronger effects

Figure 3 shows the funnel plot for RTs, and Fig. 2 the forest plot of the same dataset. The rank correlation test for funnel plot asymmetry showed significant evidence of asymmetry, suggesting potential publication bias (Kendall’s τ = 0.619, *p* = 0.0008). Additionally, a modified Egger’s test was performed, which also indicated significant evidence of publication bias (estimate = −1.2413, *p* < 0.0001), suggesting a tendency of smaller studies with less precision to report larger effect sizes. To further assess and correct for potential publication bias, a trim-and-fill analysis was conducted. This analysis estimated that four studies were potentially missing on the left side of the funnel plot (SE = 2.5999). After adjusting for these potentially missing studies, the random-effects model still showed a significant overall effect (estimate = 0.9578, 95% CI [0.5775, 1.3382], *p* < 0.0001), with substantial heterogeneity (I² = 66.56%, Q = 51.8259, *p* < 0.0001).

The fail-safe N calculations using the Rosenthal, Orwin, and Rosenberg approaches indicated that many studies with an average sample size and null result would be required to negate the observed effects. Specifically, the Rosenthal approach indicated a fail-safe N of 515, while the Rosenberg approach indicated a fail-safe N of 304. These numbers substantially exceed the threshold (5*k + 10 = 85; where k is the number of studies; Fragkos et al. 2014), above which publication bias would be minimal., suggesting robust evidence against the likelihood of publication bias undermining the findings. However, given the potentially significant implications of even small changes in RT (Jakobsen et al. 2011), we also employed Orwin’s fail-safe N with a more conservative threshold (Orwin 1983). Using a target effect size of d = 0.3, which could represent a meaningful change in RT of approximately 15ms (assuming a standard deviation of 50ms), we found that 40 studies with null results would be required to reduce the current pooled effect (d = 1.0852) to this level, suggesting that the overall effect is robust to statistical fluctuations.

### Meta analytic results: acute effects of psilocybin on accuracy

One outlier was found (Effect Size ID = 24). Running the model without this outlier did not change the direction of the effect, nor changed heterogeneity. Thus, this effect size was not excluded from the above-mentioned analyses.

**Fig. 4:**
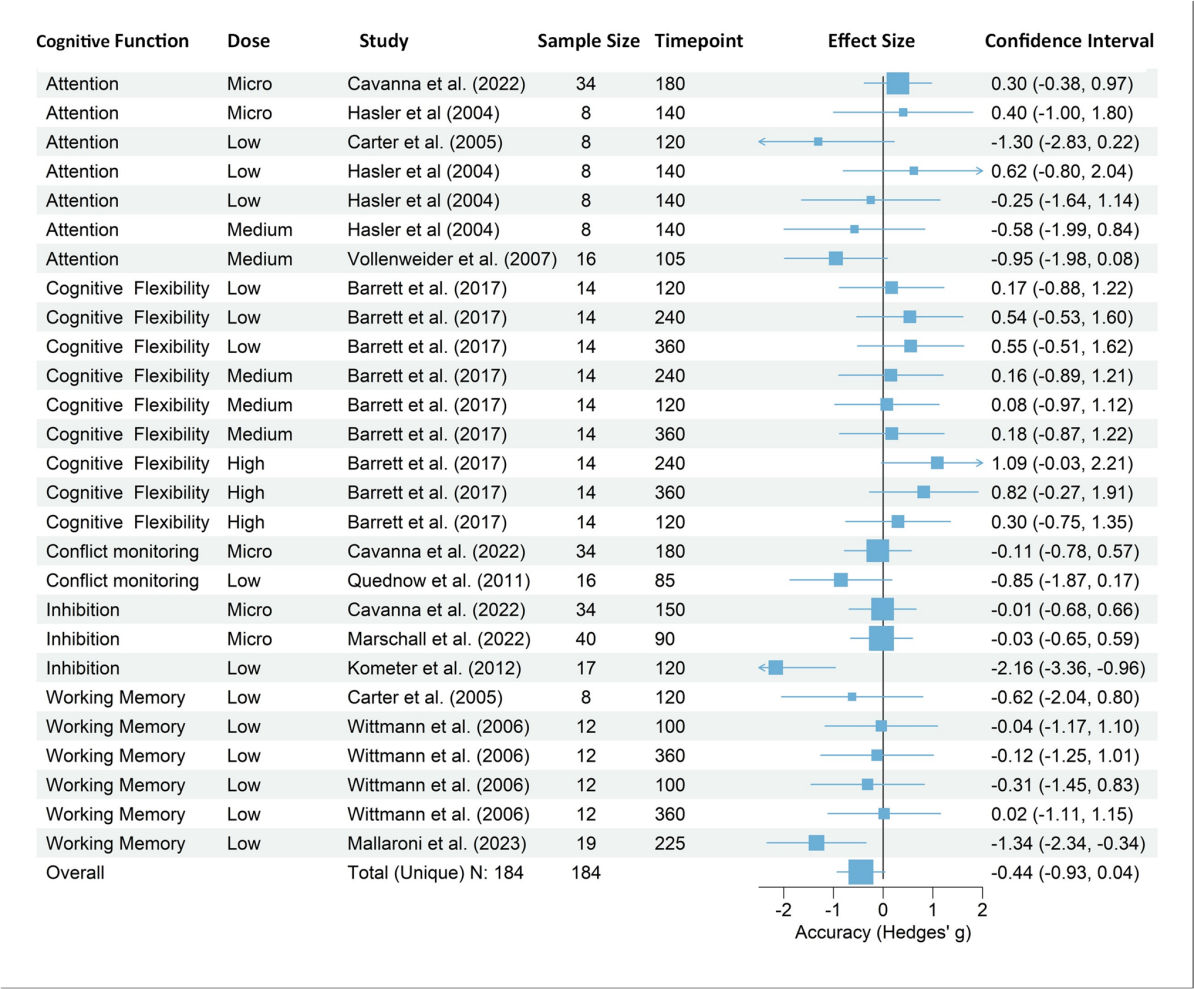
Acute effects of psilocybin on accuracy across cognitive domains and doses. Note: Forest plot of effect sizes (Hedges’ g) for psilocybin’s impact on ACC. Results are sorted by cognitive domain, showing individual study effects and the overall pooled effect. Negative values indicate decreased ACC with psilocybin compared to placebo. The size of the squares indicates the relative weight of each study, with larger squares representing larger sample sizes. Measurement timepoint is displayed minutes

A multilevel meta-analysis on the subset of 27 ACC effect sizes from 10 unique studies revealed a negative overall pooled effect size of Hedges’ g = −0.45 (SE = 0.23, t = −1.90, df = 26, *p* = 0.0681, 95% CI [−0.93, 0.034])(see Fig. 4). Heterogeneity was moderate and significant (I2 = 42.53%, Q (26) = 39.74, *p* = 0.0414). Notably, the majority of detected heterogeneity I^2^_Level3_ = 42.54%, originated from between-study differences, while no variability, I^2^_Level2_ = 0% was attributed to within-study differences.

A between-study heterogeneity variance of τ²(Level3) = 0.39 and no within-study variance (τ²(Level2) = 0)) was observed. The absence of within-study variance might indicate that the variability within individual studies (e.g., due to measurement error or within-study sampling variability) is negligible. This could imply that the effect sizes from individual studies are very consistent.

The comparison of the nested model with the non-nested model revealed that the nested model was statistically superior (𝝌12 = 10.33, *p* = 0.0013), as indicated by its lower AIC and BIC values (Full model AIC = 50.3, BIC = 54.01; Reduced model AIC = 58.63, BIC = 61.14), suggesting that the nested model provides a better fit by effectively capturing additional variability.

#### Moderators of Accuracy Effects

Subgroup analyses were conducted to explore potential moderators of psilocybin’s effect on ACC. The timing of psilocybin administration (peak vs. non-peak) did not significantly moderate the effect (QM (1) = 0.52, *p* = 0.47). Dosage categorization (micro, low, medium, high) also did not yield significant moderation (QM (3) = 4.38, *p* = 0.22), nor did a simplified micro/low vs. medium/high comparison (QM (1) = 0.02, *p* = 0.90). Cognitive function categories (attention, conflict monitoring, other executive functions, working memory) showed no significant moderation effect (QM (2) = 0.19, *p* = 0.91). In line with these observations, a metaforest machine learning algorithm also did not reveal a sufficient fit of the moderation model with the mentioned variables (see supplementary material).

These results indicate that while overall heterogeneity was observed, our tested moderators did not significantly explain this variability in psilocybin’s effects on ACC.

#### Evaluation of publication bias in accuracy studies

**Fig. 5:**
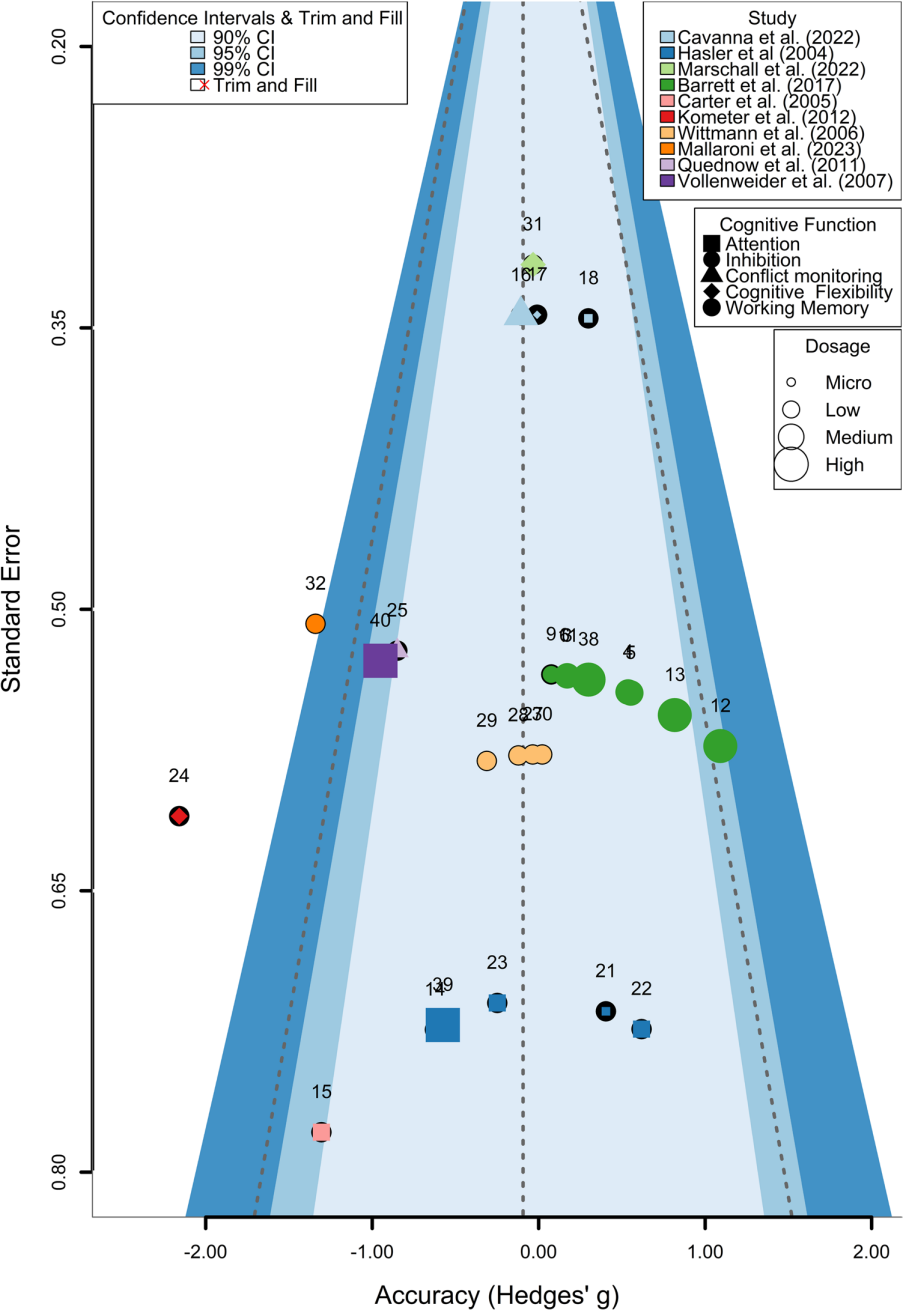
Publication bias assessment for psilocybin’s effects on accuracy. Note: Funnel plot illustrating the effects of psilocybin on ACC across various studies. Each point represents an individual study outcome, with different shapes indicating cognitive functions, colors denoting studies, and sizes reflecting dosages. Shaded areas represent 90%, 95%, and 99% confidence intervals. The plot does not indicate the presence of publication bias

Figure 5 shows the funnel plot for ACC, and Fig. 4 the forest plot of the same dataset. In the accuracy dataset, the rank correlation test for funnel plot asymmetry was not significant (Kendall’s τ = −0.05, *p* = 0.74). The Fail-safe N calculations did not indicate the presence of publication bias. In addition, a modified Egger’s test was conducted to further assess the potential for publication bias. The Egger’s test, which uses precision as a moderator in a meta-analysis framework, did not reveal significant evidence of publication bias (QM = 0.64, df = 1, *p* = 0.42). This suggests that the effect sizes in the analysis are not disproportionately influenced by study size or precision, a common indicator of publication bias.

### Risk of bias assessment results

**Fig. 6:**
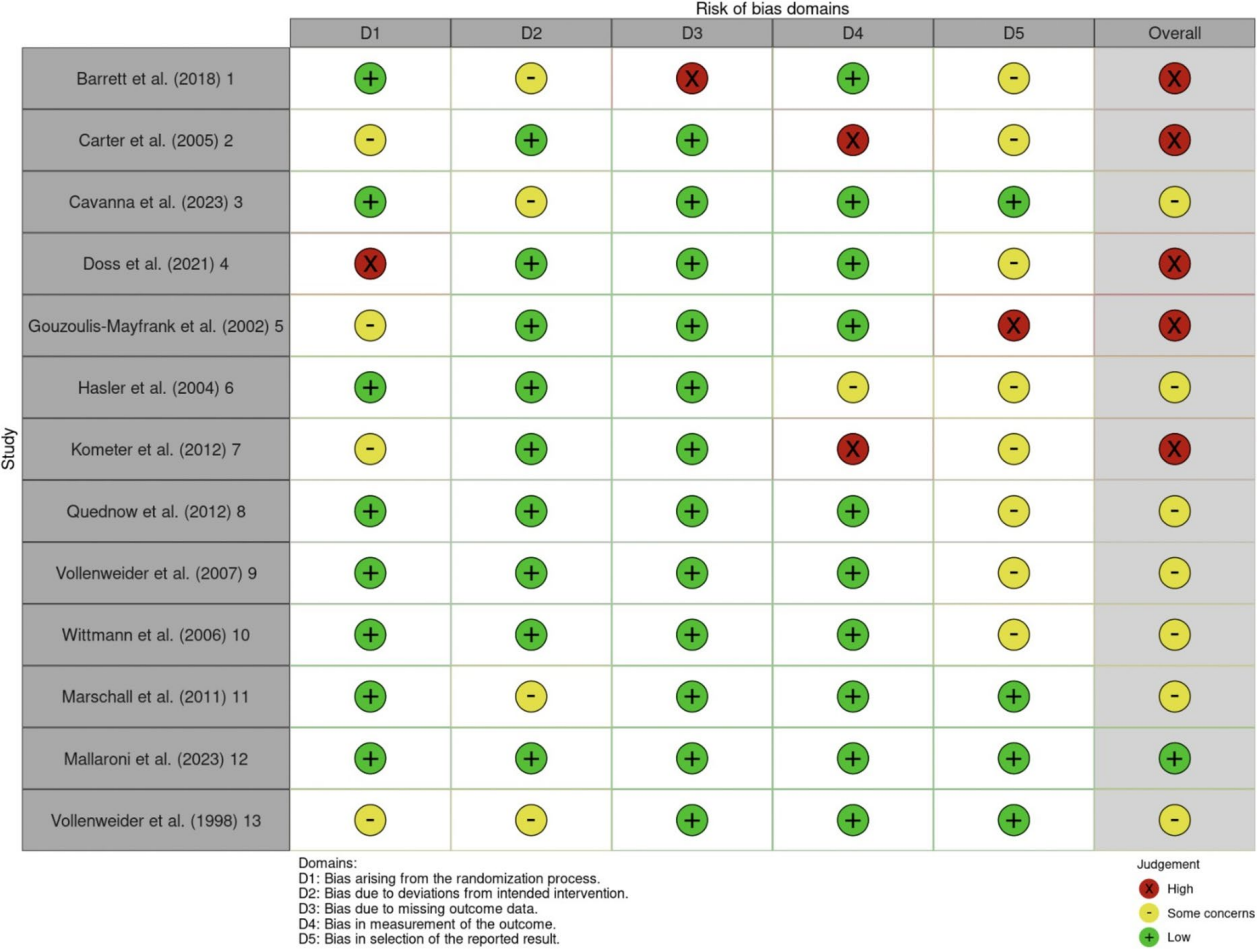
Risk of bias assessment for individual studies. Note: Heat map showing risk of bias assessments for 13 studies across five domains (D1-D5) and overall bias. Green (+) indicates low risk, yellow (-) indicates some concerns, and red (X) indicates high risk of bias. Domains assessed include randomization process (D1), intervention (D2), missing outcome data (D3), outcome measurement (D4), and selection of reported results (D5). The study ID is indicated after the study citation in the first column

**Fig. 7:**
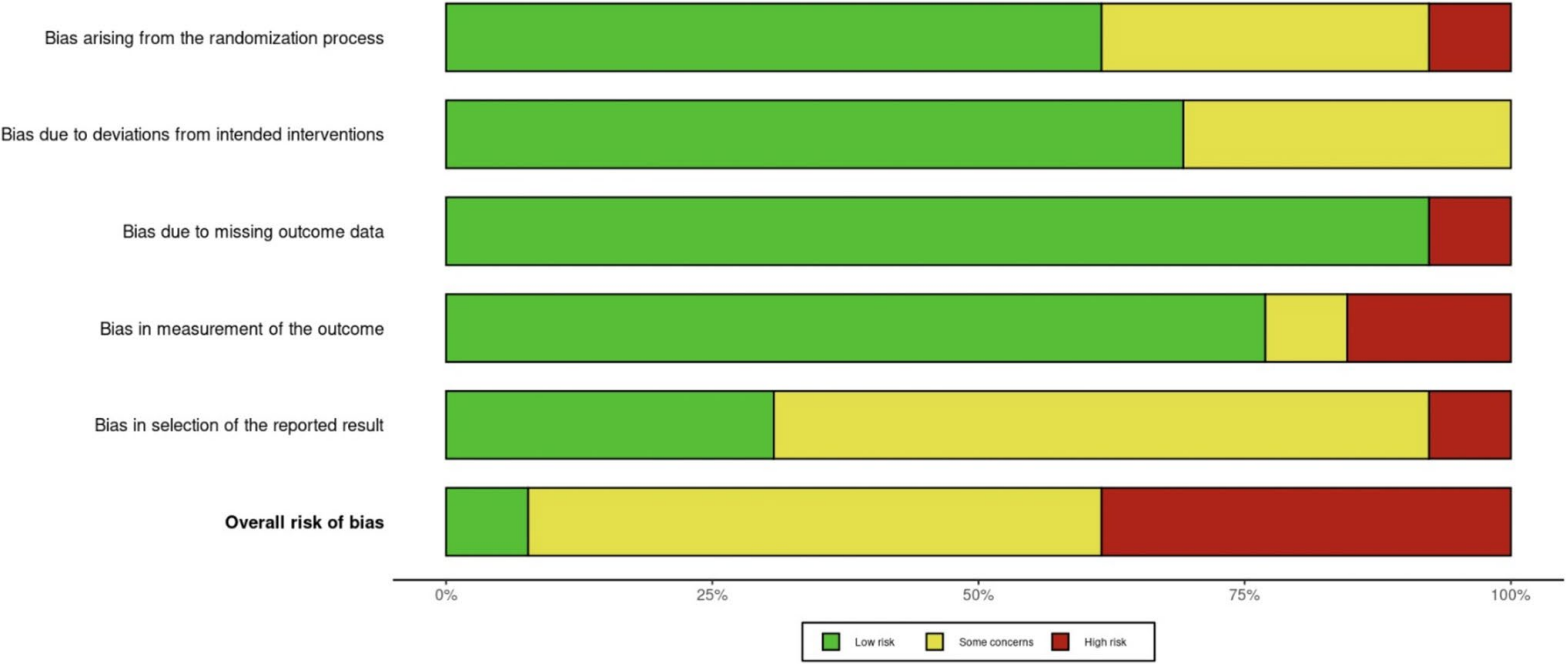
Overall risk of bias across studies. Note: Summary of overall risk of bias assessments for included studies. The chart displays the proportion of studies classified as having low risk, some concerns, or high risk of bias

 An overview of the risk of bias assessment is displayed in Figs. 6 and 7. The interrater reliability analysis yielded evidence of good agreement between raters as to the presence of bias in the studies (i.e., both raters marked some or high concern) Kappa = 0.629, z = 1.165, *p* = 0.247. However, poor interrater reliability was illustrated (QM (3) = 2.96, *p* = 0.40). Similarly, executive function task sensitivity levels did not significantly moderate the effect for the level of bias (i.e., both raters put “some concerns” as opposed to “high concerns”) Kappa = 0.170, z = 0.687, *p* = 0.492. Figure 7 contains the consensus combination chart illustrating level of bias between domains for each study.

## Discussion

Despite the growing interest in psilocybin’s therapeutic potential, its’ acute effects on cognition have not yet been systematically investigated. This paper addresses that gap through a comprehensive systematic review and meta-analysis. After initial abstract screening, 42 effect sizes from 13 individual studies were extracted and categorized into different domains of executive functions and attention. Importantly, the overall risk of bias across the studies included in our analysis is moderate to high. Most noticeably, this is driven by concerns about blinding procedures as well as lack of pre-registrations. Additionally, our investigation points towards a publication bias for reaction time (see Fig. 3), but not accuracy (see Fig. 5), given the asymmetry of the funnel plots. In the original studies included in the meta-analysis, cognition was often of secondary interest, which poses the question, whether other research groups failed to report their non-significant results, as they were also not primarily interested in cognition, thus driving the publication bias. Consequently, the heterogeneity, risk of bias, and potential for publication bias could lead to an overestimation of the true effect size within our analysis, skewing the data towards significant findings.

In our meta-analysis we found that psilocybin acutely reduces ACC slightly to moderately, albeit non-significantly, and largely slows RTs in cognitive tasks assessing executive functions and attention. We further found that this effect on RT was significantly moderated by (i) dosage (micro, small, mid, high), in that higher doses more strongly impacted RTs; and (ii) measurement sensitivity (general, specific, pure), in which more general measures showed larger effects. No significant moderation has been observed in (iii) subcomponents of executive functions and attention (working memory updating, inhibition, multiple executive functions, attention) and (iv) time point of measurement (during peak, after peak). Due to the non-significant overall effect on ACC, none of the moderators mentioned above reached significance and will thus be disregarded for further discussion.

### Moderation effects of reaction time

#### The influence of dose on reaction time

First, the effect of psilocybin on RT slowing on executive functions and attention follows a linear dose-dependent relationship, with higher doses showing a stronger slowing of RTs, and lower doses having less impact. Given that psilocybin has a dose response effect on psilocin plasma concentration (Holze et al. 2023), and subjective experience ratings (Hirschfeld and Schmidt 2021), it is not surprising that this trend is present for performance in executive functions and attention as well. This dose-dependent effect is observed in all four studies that investigated different dosages. Interestingly, both studies investigating working memory (updating) (Wittmann et al. 2006; Barrett et al.^2018^), showed significantly slower RTs at high dose, but not at medium dose compared to placebo. However, studies investigating attention (Hasler et al. 2004; Vollenweider et al. 2007) found reduced performance already at both low and medium dosages. This suggests that while generally there is a dose-dependent effect of psilocybin across cognitive functioning, specifically executive functions, such as working memory updating might be slightly more resilient for these effects.

#### The influence of timing on reaction time

In the included studies, executive functions and/or attention were measured between 60 and 240 min post-psilocybin administration, out of which seven effect sizes were obtained during the peak window (90–180 min post ingestion; Barrett et al. 2018; Cavanna et al. 2022; Kometer et al. 2012; Mallaroni et al. 2023), and eight outside the peak window (> 90 and ,<180 min post ingestion) of the psilocybin drug effect (Barrett et al. 2018; Cavanna et al. 2022; Gouzoulis-Mayfrank et al. 2002; Quednow et al. 2011; Vollenweider et al. 1998). In contrast to dose as a moderator, the measurement timepoints did not significantly influence the effects of psilocybin on RT. Although our study differentiated between measurements taken within the peak window (90–180 min post-ingestion) and those taken outside it, all measurement time points fall within the acute phase of psilocybin’s effects. The data indicate that the effects are consistently distributed throughout this window, irrespective of whether they occur within or outside the peak window. To establish a robust dose-response curve for psilocybin’s effects on cognition, further studies incorporating a substantially wider range of acute, post-acute and long-term timepoints are necessary.

#### The influence of measurement sensitivity on reaction time

Observations from the primary studies varied widely due to differences in measurement techniques and statistical methods, prompting us to examine if the granularity of these measures affected reported effects on RTs. This moderation analysis revealed that the degree of measurement sensitivity moderates the effect on RT, with more general measures of sensitivity showing a stronger effect than more specific measures. This suggests that psilocybin’s impact on RT is more general rather than specific to executive functions, as more specific methods aim to account for general function by, for example, calculating a difference score. For instance, in the Stroop task, the congruent and incongruent conditions both necessitate similar levels of basic sensory processing and motor responses (Adleman et al. 2002); however, they vary in the extent to which they engage cognitive control and conflict monitoring, thereby partially isolating the cognitive domain of interest if the scores are subtracted from each other. The fact that measurement sensitivity is a significant moderator, indicates that a significant amount of the effect could be attributed to the underlying general functions such as psychomotor speed and/or attention, rather than the specific cognitive domain. This suggests that the lower level cognitive and motor functions involved in these tasks could play an important part in the observed RT slowing, on top of the specific cognitive domains targeted by the more precise measures.

#### The influence of subcomponents of executive functions and attention on reaction time

We further investigated whether the RT slowing effects of psilocybin vary across subcomponents of executive functions and attention. Our analysis revealed that the effects were not specific for specific subcomponents, suggesting that psilocybin acutely affects executive functions and attentional abilities in a similar manner. This points to a potential mechanism being affected by psilocybin that equally impacts all assessed cognitive domains.

### Reaction time and accuracy

**Fig. 8:**
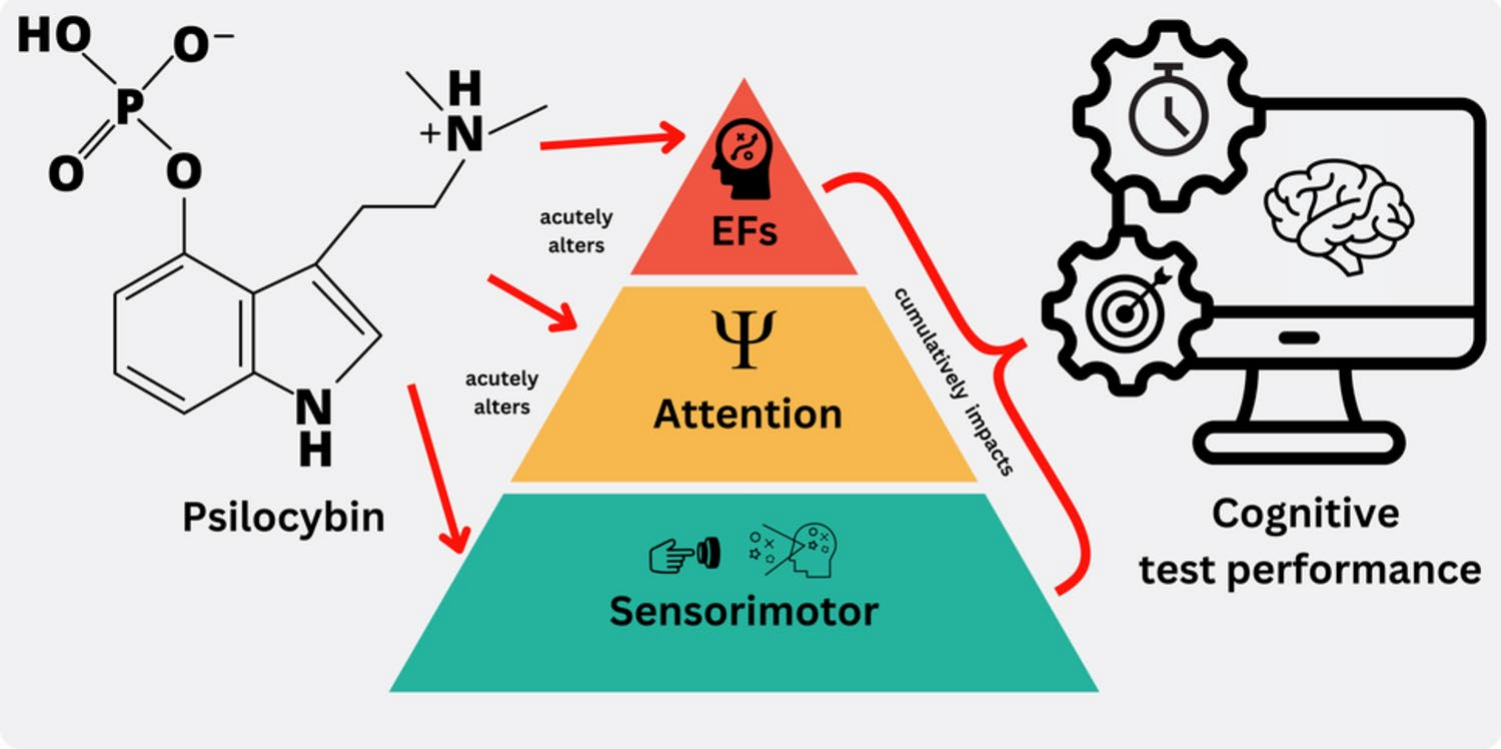
Multi-level explanation of the acute effects of psilocybin on reaction time and accuracy in executive function and attention tasks. Note: This figure illustrates a theoretical multilevel model delineating the acute effects of psilocybin on cognitive test performance, specifically reaction time and accuracy. Psilocybin may acutely alter executive functions, attention, as well as sensorimotor functions. These influences can occur independently at each level or interact synergistically, ultimately resulting in a negative impact on overall task performance

The data suggest that psilocybin slows RT in executive functioning and attention tasks in a dose dependent manner, while the effects on ACC are not that clear. Although the cognitive tasks included in this analysis aim to isolate specific cognitive domains, overall performance is inevitably influenced by a variety of additional functions. These include more basic processes such as motor preparedness and psychomotor speed (involved in executing a button press), attentional capabilities (e.g., to what extent the participant adhered to the instructions), and higher-level executive functions such as task switching abilities, and motivation. Therefore, below we discuss our findings within a framework of a multilevel explanation and propose potential mechanisms through which psilocybin may lead to these outcomes by impacting various cognitive levels separately or simultaneously (see Fig. 8). It is important to note that this interpretation is speculative and should be used to form new, testable hypotheses for future research.

#### Effects of psilocybin on cognitive task performance: sensorimotor functions

As the motor cortex is responsible for the execution of voluntary movements, modulation of 5HT-2a receptor activity through psilocybin could affect motor cortex excitability and thus impact RTs in cognitive tasks by slowing psychomotor speed, as demonstrated by Wittmann et al. (2006), and Barrett et al. (2018). Other basic functioning areas might be involved in the generalized slowing of RT as well, for example alterations of the sensitivity of the visual system. Several studies suggest that psilocybin and other 5HT-2a receptor agonist inhibit connectivity within the visual pathway, potentially reducing its responsiveness, thus slowing the overall processing speed (Stoliker et al.^2024^ Azimi et al. 2020; Evarts et al. 1955; Michaiel et al. 2019).

Taken together, modulation through psilocybin within the motor system as well as the visual pathways might explain parts of the RT slowing observed in our data.

#### Effects of psilocybin on cognitive task performance through attention

While part of the effects of psilocybin on RT might be driven by alterations in basic functions such as motor and visual performance, other parts, particularly the effects on ACC, may be explained through psilocybin’s effect on attention. Since attention is a fundamental building block for higher cognitive functions (Burgoyne and Engle 2020; Rose et al. 2003), psilocybin’s impact on attentional processes could significantly influence cognitive task outcomes, which may explain the patterns observed in our data. When attention is compromised, individuals find it more difficult to maintain focus, adhere to task instructions, or manage distractions effectively, leading to poorer performance across various tasks regardless of their specific demands (Prinzmetal et al. 2005). Indeed, Vollenweider et al. (2007) demonstrated significant reductions in performance on the FAIR task, which assess attentional capacity across low, medium, and high doses of psilocybin, during both the peak and post-peak drug effect. This suggests a global negative impact on attentional performance, a trend similarly observed in Hasler et al. (2004), who also used the FAIR task. Furthermore, Carter et al. (2005) reported that attentional tracking was adversely affected by psilocybin under low doses during an attentional object tracking task, indicating that even lower doses of psilocybin can impair attentional capacities. Reduced attentional capabilities do not only lead to slower RTs, but also to reduced ACC (see Chen et al. 2022 for recent meta-analysis). This suggests that attention may be a key factor in the observed effects of psilocybin on task performance on most (if not all) cognitive tests included in our analysis, as general performance is dependent on attentional capabilities.

#### Effects of psilocybin on cognitive task performance on executive functions. Dual-task and motivation

A third mechanism underlying the observed effects of psilocybin on reactions and ACC can be found in the dual-task nature of cognitive-experimental studies with psychedelics. The dual-task interference theory posits that the processing of multiple interfering tasks leads to a cognitive cost, manifesting in slowed RTs and drops in ACC (Koch et al. 2018; Wickens 2002; Stets et al. 2019; Kiesel et al. 2010; Leone et al. 2017). During the acute phase of psilocybin, managing the intense subjective experiences of the psychedelic trip could be considered a cognitive task in and of itself, demanding significant cognitive resources, which could lead to effects similarly observed in studies investigating dual-task effects. In particular, dual-tasking slows RTs and decreased ACC due to rapid task switching and cognitive recalibration (for review see Koch et al. 2018).

These cognitive recalibrations —ignoring interferences, implementing new task rules, and updating working memory (Burgess and Shallice 1996; Shallice and Burgess 1991; Burgess et al. 2000; Strayer and Johnston 2001; Chen and Hsieh 2023; Wylie and Allport 2000; Monsell, 2003; Kieffaber and Hetrick 2005; Snyder et al. 2020) —are heavily reliant on cognitive control (Egner 2023; Meiran 2000). Cognitive control, which is critically supported by the Claustro-Cortical-Circuit (CCC) Network, plays a crucial role in efficiently guiding attention towards task-relevant stimuli while suppressing distractions (Lavie 2010; Miller 2000; O’Reilly et al. 2010). While empirical evidence is limited, one study suggests that psilocybin may transiently disrupt these higher-level cognitive control mechanisms through 5HT-2a receptor-mediated desynchronization within the CCC Network, including the highly interconnected claustrum (Barrett et al. 2020a, b; Doss et al. 2022). Such disruption could impair cognitive control, making it more challenging to manage dual-task demands, and thus contributing to the observed decreases in task performance under psilocybin.

Participants’ accounts further illustrate the potential challenges, that arise in these conditions. The profound and captivating nature of the psychedelic experience often creates a mismatch between what participants find meaningful and the tasks expected by experimenters, thus interfering with the primary task (for discussion, see: Langlitz 2013). For example, McCulloch et al. (2021) reported a participant from an LSD trial expressing a deep existential insight yet feeling constrained by the mundane requirement to “look into a TV-screen.” Similarly, Robinson (1966) noted a participant’s disinterest in test stimuli, stating a desire to immerse themselves in the experience rather than perform experimental tasks. These accounts illustrate that the intense nature of these experiences not only imposes substantial cognitive demands, potentially causing a dual-task-interference-like cost, but may also diminish participants’ motivation to fully engage with the primary task, as they are perceived as less meaningful and more effortful than the psychedelic experience.

Typically, low ecological validity is the reason for this problem (Robertson and Schmitter-Edgecombe 2017), and more naturalistic tasks, such as assessing attention and executive functions during music performance or listening under the influence of psilocybin, could potentially show the opposite effect. These tasks may not only be more motivating for participants due to their real-world relevance, but could act in synergy with the psychedelic experience rather than in competition, thus preventing dual-task interference cost, and motivational issues.

In short, we argue that basic sensorimotor processing, attention and dual-task performance and motivation all could account for the detrimental effects we observed for psilocybin in RTs and ACC.

### Recommendations for future assessment of cognition under the influence of psychedelics

To directly assesses some of the mechanisms that drive the observed effects, a thought-probe mind-wandering paradigm (Franklin et al. 2011) could be employed to evaluate dual-task interference and attention. This method would allow to assess if participants are more readily distracted by their psychedelic experiences and whether psilocybin increases the frequency of off-task thoughts and experiences, leading to greater impairments in cognitive performance.

Alternatively, novel test paradigms could be used instead of traditional cognitive tests as the latter suffer from low ecological validity and may not fully capture the broad and overlapping effects of psilocybin on multiple levels. Assessing psilocybin’s effects on executive functions and attention in naturalistic settings, as mentioned above, might reveal enhancements in some domains, as anecdotal reports sometimes denote improved motor skills, like juggling or states of heightened attention whilst listening to music (Day and Schmetkamp 2022). Another direction could be the incorporation of no-response or task-free paradigms (e.g. eye-tracking or experience sampling), which can provide insights into cognitive function without relying on conventional task performance measures (for review see Duman et al. 2022; Baror and He 2021).

### Lack of long-term assessment of psilocybin on executive functions and attention

A major limitation of this meta-analysis is that the results focus exclusively on the acute effects of psilocybin on cognition, as there were almost no studies with measurements at later time points that met our inclusion criteria. The only exception was the previously discussed study by Doss et al. (2021) where improvements in cognitive flexibility were observed up to one month after the treatment (Doss et al. 2021), which lasted up to a year for the majority of the participants (Gukasyan 2022). Further, a recent large scale self-report based longitudinal study involving 2,503 older adults (average age 64 ± 11 years), showed that psychedelic use within the 12 months prior to assessment was related to faster RT and increased ACC in an executive function task battery in addition to fewer depressive symptoms, although no similar effect was observed for episodic memory (Fearn and Bhattacharyya 2024). This goes to show that contrary to the acute slowing of RTs and reduced ACC under the influence of psychedelics observed in our study, more recent studies are pointing towards potential long-term cognitive benefits. Some studies suggest that these long- term changes might be mediated and facilitated by an increase in neuronal plasticity after psychedelics use (for review see Calder and Hasler 2023), but to our knowledge, the relationship between psychedelic-induced neuroplasticity and cognitive performance has yet to be investigated. Thus, further research is needed to establish a clearer picture on the mechanisms that guide the acute functional impairments of psilocybin, as well as potential long-term benefits of using psilocybin.

### Implications of the meta-analytic results on safety

The results of our meta-analysis underscore the necessity for adequate supervision in therapeutic and recreational settings where psilocybin is being used. Although the negative impact on cognition is most likely transient, the exact duration of this effect remains unclear. Thus, it is particularly relevant for patients and participants in psychedelic studies to avoid potentially hazardous situations, such as participating in traffic or operating heavy machinery, during the acute phases and the following days of psilocybin. Especially, considering the increased acceptability and use of psychedelics for treatment or recreational purposes, to reduce harm it is imperative for future studies to assess the impact and timeframe of these side effects more systematically. One exemplary tool in this regard is the newly developed Swiss Psychedelic Side Effect Scale, which is designed to address this need comprehensively (Calder and Hasler 2024).

## Conclusion

The current exploratory meta-analysis demonstrates that psilocybin generally slows RTs in cognitive tasks assessing executive functions and attention, with a clear dose-dependent effect, where higher doses result in more substantial slowing. This effect is significantly moderated by the sensitivity of the measurement, indicating that general measures are more sensitive to the impact of psilocybin than specific ones. Even though there is a small to moderate effect of psilocybin on ACC as well, these findings are less consistent. The lack of significant moderation by subcomponents of executive functions and attention suggests that psilocybin’s effects are more generalized across cognitive domains. We argue that the observed psilocybin-induced slowing of RTs is likely mediated by basic processes including (1) basic sensorimotor processing, (2) attentional impairments and (3) task-switching and motivation.

Our analysis is constrained by moderate to high risk of bias across the included studies, notably due to concerns regarding blinding procedures and a lack of pre-registrations. There was also a potential for publication bias for RT, as the asymmetry of the funnel plot suggests that non-significant results may either have been underreported, or that the data is skewed due to smaller studies having larger effect sizes. This could lead to an overestimation of the true effect size, as the bigger effects reported could be less precise due to small samples.

In conclusion, while psilocybin negatively affects cognitive task performance acutely, the exact nature and mechanisms of these effects require further elucidation. The findings of this meta-analysis should inform future research, which can aim to unravel both the immediate and enduring impacts of psilocybin on the brain and cognition.

## Further information

### Registration and protocol

For the present study, no prior hypotheses were set. Furthermore, the present study was not pre-registered, and also no study protocol was produced prior to data collection and analysis, thus, the present study is considered exploratory.

*From*: Page MJ, McKenzie JE, Bossuyt PM, Boutron I, Hoffmann TC, Mulrow CD, et al. The PRISMA 2020 statement: an updated guideline for reporting systematic reviews. BMJ 2021;372:n71. doi: 10.1136/bmj.n71.

For more information, visit: http://www.prisma-statement.org/.

## Supplementary Information

Below is the link to the electronic supplementary material.

Supplementary Material 1(DOCX 672 KB)

## Data Availability

All the data and code, as well as further analyses underlying the present study are available *here* as supplementary material. We adhere to OPEN guidelines and encourage the usage of our data for further exploration.
