# Supplementary material for: Acute effects of psilocybin on attention and executive functioning in healthy volunteers: a systematic review and multilevel meta-analysis
Source: Psychopharmacology (Berl). 2025 Jan 23;242(6):1171–96. doi: 10.1007/s00213-024-06742-2 (PMC12084245; doi:10.1007/s00213-024-06742-2)
Supplement: Supplementary file 1 — Supplementary Material 1(DOCX 672 KB) [file 213_2024_6742_MOESM1_ESM.docx]

**Supplementary Results**

**Acute Effects of Psilocybin on Attention and Executive Functioning in Healthy Volunteers: A Systematic Review and Multilevel Meta-Analysis**

Yousefi, P.^a*^ , Lietz, M.P. ^b,c*^, O’Higgins, F. J.^d^,  Rippe, R.C.A.^e^, Hasler, G.^b,f,g^, van Elk, M.^a^, Enriquez-Geppert, S.^c,g,f^

^a^ Cognitive Psychology Unit, Institute of Psychology, Leiden University, the Netherlands

^b^ University Center for Psychiatric Research, University of Fribourg, Villars-sur-Glâne, Switzerland

^c^ Department of Clinical and Developmental Neuropsychology, University of Groningen, Groningen, the Netherlands

^d^ Royal College of Surgeons in Ireland, Dublin, Ireland

^e^ Institute of Education and Child Studies, Leiden University, the Netherlands

^f^ Freiburger Netzwerk für psychische Gesundheit, Villars-sur-Glâne, Switzerland

^g^ Lake Lucerne Institute, Vitznau, Switzerland

^i^ Department of Biomedical Sciences of Cells & Systems, University Medical Center Groningen, Groningen, the Netherlands

^j^ Psychedelic Treatment and Mechanisms Group, University Centre of Psychiatry, Groningen, the Netherlands

^*^These authors contributed equally.

**Submitted to *Psychopharmacology***

**Correspondence:**
Morten Lietz
Email: [morten.lietz@unifr.ch](mailto:morten.lietz@unifr.ch); mortenlietz@gmail.com

**Meta Forest Analysis**

To address between-studies heterogeneity and identify relevant moderators without overfitting or assuming linearity, we employed the MetaForest technique (Van Lissa, 2017). This machine learning approach, based on random forests (Strobl et al., 2009), performs variable selection in meta-analyses. Our dependent variables were the Cohen's d effect sizes for accuracy (ACC) and reaction time (RT), calculated as the difference between psilocybin and placebo conditions. Predictors included cognitive function category, dosage, administration timing, and the sensitivity of measures to executive functions (EF sensitivity). We initially ran 20,000 trees to determine convergence (Figure S1), then refined to 5,000 trees for computational efficiency. Moderators were retained if they showed positive variable importance in over 50% of 300 replications using a recursive pre-selection method. This approach allowed us to assess how these factors moderated psilocybin's effects across studies, providing the exploration of influential moderators without the constraints of traditional linear models.

***Reaction Time***

Our fine-tuned MetaForest model for RT demonstrated good predictive performance, with an out-of-bag $R^{2}OOB$ of 0.4991 and a cross-validated R²CV of 0.5879. This indicates that approximately 49.91% to 58.79% of the variance in the RT data could be explained by the model. The analysis confirmed the substantial influence of Executive Function (EF) sensitivity and Cognitive Function, as shown in the variable importance plot, suggesting these factors play crucial roles in moderating the effects of psilocybin on RT (Figure S2). The residual heterogeneity (tau²) of 0.0863 indicates that while our model explains a significant portion of the variance, there is still some unexplained variability in the effects across studies.

Further analysis revealed that psilocybin's effect on RTs increases with increasing Executive Function task sensitivity (see Figure S3). Specifically, the influence of psilocybin is most pronounced in tasks least sensitive in capturing the effect on executive functions (EF_sensitivity level 3), with level 3 showing the strongest effect. Among specific cognitive functions, working memory and cognitive flexibility tasks were most affected, while attention tasks were least impacted.

Moderated partial dependence plots (Figures S4) further illustrated that lower sensitivity of Executive Function tasks (higher values of EF_sensitivity) were associated with slower RTs, particularly in tasks involving cognitive flexibility and conflict monitoring. This underscores the role of executive function task sensitivity in capturing psilocybin's effects, demonstrating a clear gradation where increased sensitivity levels correspond to stronger effects of psilocybin on RTs.

***Accuracy***

Our fine-tuned MetaForest model for ACC demonstrated poor predictive performance, with an out-of-bag $R^{2}OOB$ of -2.115 and a cross-validated R²CV of 0.263. This indicates that the model performed worse than a null model, explaining none of the variance in the ACC data, but with cross-validation showing some predictive capability at 26.3%. The analysis confirmed the substantial influence of dosage, as shown in the variable importance plot (Figure S2). The residual heterogeneity (tau²) of 0.9740 indicates a high level of unexplained variability in the effects across studies. Further analysis revealed that psilocybin's effect on ACC is influenced by the dosage levels (Figure S3). Specifically, low and medium doses tend to result in lower ACC outcomes compared to high and micro doses. Moderated partial dependence plots (Figures S5) further illustrated the complex, non-linear relationships between dosage levels and cognitive functions on ACC outcomes. These plots highlight that the interaction between different cognitive functions and varying doses of psilocybin can lead to significant differences in ACC, indicating that the relationship between psilocybin dosage and cognitive performance is not straightforward. In general, across all cognitive function categories, micro doses had consistently the highest impact on ACC.

**P & Z Curve Analysis**

In this study, we employed both Z-curve and p-curve analyses to assess the replicability, discovery rates, and evidential value of our findings. Z-curve, implemented using the zcurve package in R (Bartoš & Schimmack, 2022), corrects for selection bias and provides quantitative estimates of the observed discovery rate (ODR) and the expected discovery rate (EDR). Additionally, p-curve analysis (Simonsohn et al., 2013) examines the distribution of p-values from independent studies to determine whether the significant results are likely due to true effects or selective reporting. The results of this analysis for RT are displayed in Figure S6, and for ACC in Figure S7.

The z-curve analysis of RT data (N = 15 studies) revealed an Expected Replication Rate (ERR) of 84.9%, 95% CI [48.6%, 88.1%], and an Expected Discovery Rate (EDR) of 84.7%, 95% CI [13.4%, 90.1%]. The Observed Discovery Rate (ODR) was 67%, 95% CI [39%, 87%]. A chi-square test indicated that the distribution of p-values (of the differences in RT and ACC between the psilocybin and control condition) differed significantly from the null hypothesis, χ2(4) = 23, p < .001. The p-curve was skewed, with most significant p-values falling in the 0.00-0.01 range. The estimated file drawer ratio was 0.180, suggesting approximately 0.18 unpublished studies for every published study. Based on these results, the RT effects appear to have strong evidential value and a high likelihood of replicability, as indicated by the high Expected Replication Rate and Expected Discovery Rate. The skewed p-curve further supports the presence of genuine effects rather than p-hacking, though the discrepancy between the EDR and ODR suggests there may be some degree of publication bias or file drawer effect present in the literature.

The p-curve analysis for ACC data revealed only 2 statistically significant p-values, both falling in the 0.00-0.01 range. A chi-square test indicated that this distribution did not significantly differ from the null hypothesis expectation, χ2(4) = 8, p = .092. The z-curve analysis could not be performed due to insufficient significant results. These results suggest limited evidence for robust effects in the ACC data. The scarcity of significant p-values prevents a comprehensive assessment of evidential value or replicability. While the two significant results are in the strongest p-value range, the overall pattern does not provide strong support for widespread true effects in ACC measures. The inability to conduct a z-curve analysis further limits our capacity to estimate replication rates or assess publication bias. These findings indicate that caution should be exercised when interpreting ACC effects in this context.**Figure S1: Convergence plots for Reaction Time and Accuracy Metaforest**
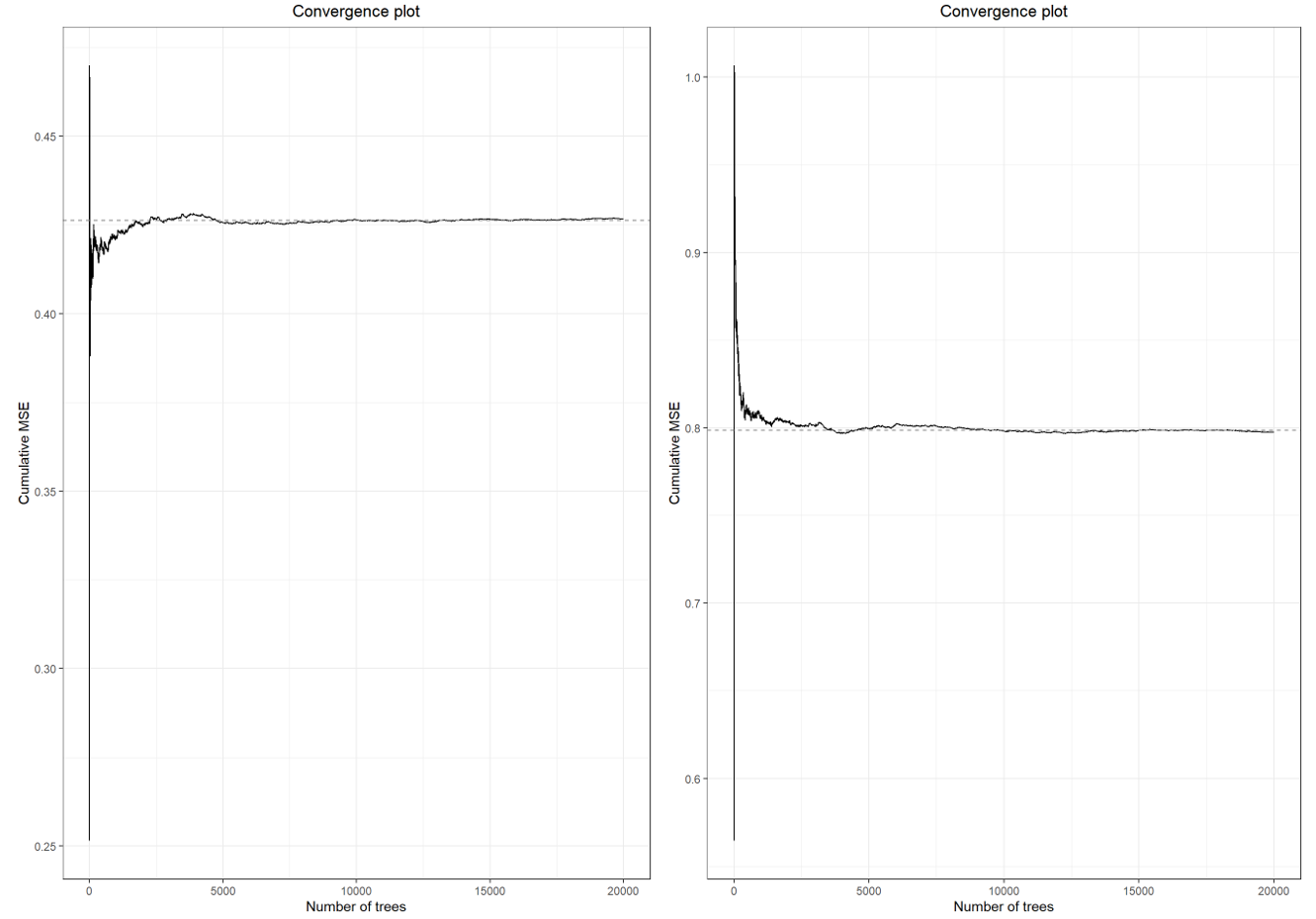


*Note.* Convergence plots for MetaForest analyses of RT (left) and ACC (right) measures in the psilocybin and cognition meta-analysis. The graphs show the generalization error over the number of trees grown. For RT, convergence is achieved rapidly and remains stable, while for ACC, the error decreases more gradually before stabilizing. Both converge at around 5000 trees.

**Figure S2: Recursive Variable Importance Reaction Time and Accuracy**

**
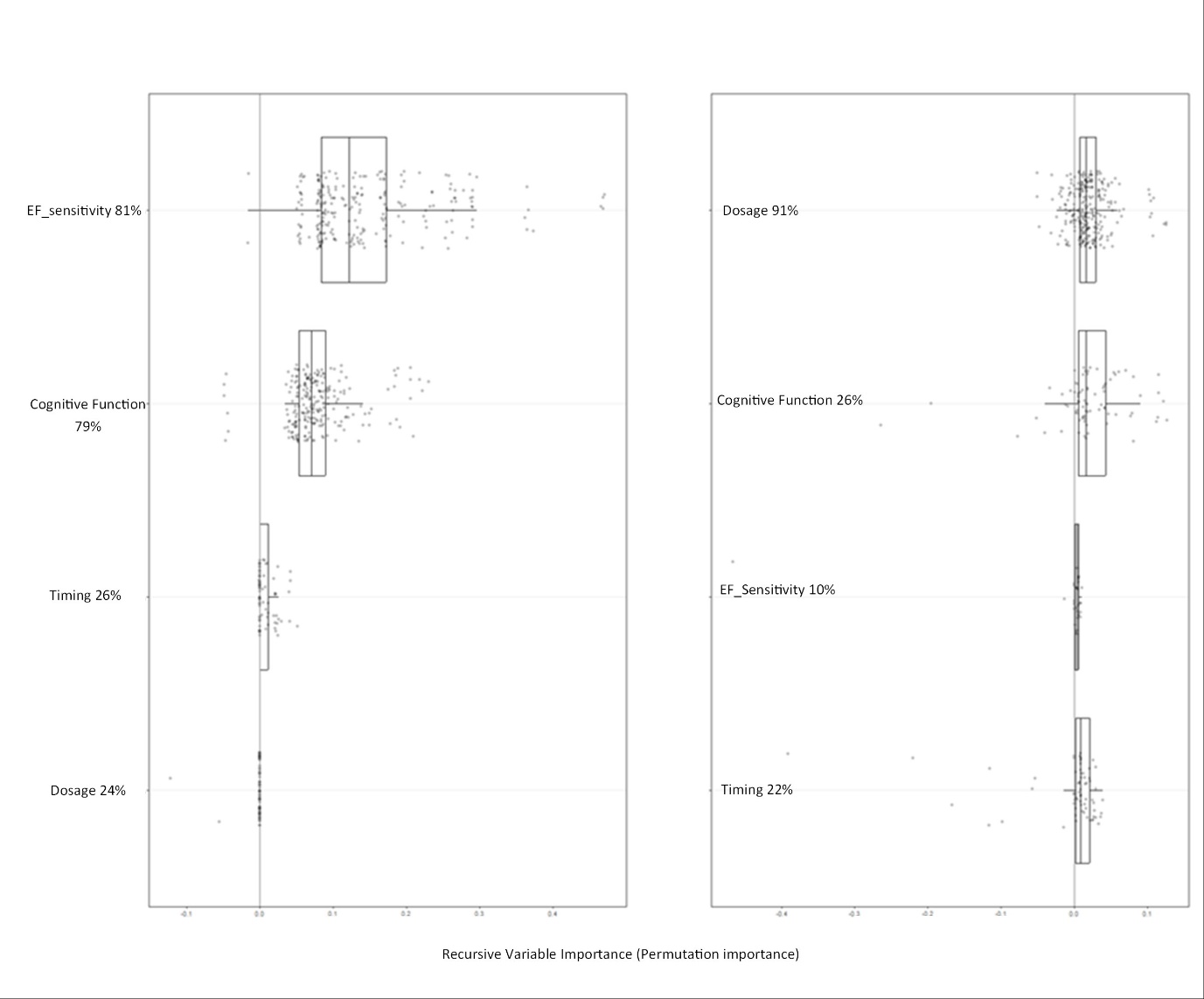
**

*Note*. Recursive Variable importance plots for RT (left) and ACC (right). The plots show the relative importance of different moderators in explaining heterogeneity across studies. For RT, EF sensitivity appears to be the most important variable, followed by cognitive function. For ACC, dose label emerges as the most important variable. The x-axis represents the relative variable importance (permutation importance), while the y-axis lists the moderator variables. Boxplots indicate the distribution of importance scores across multiple iterations (300), with dots representing individual data points.

**Figure S3: Partial Dependence Plots Reaction Time and Accuracy**
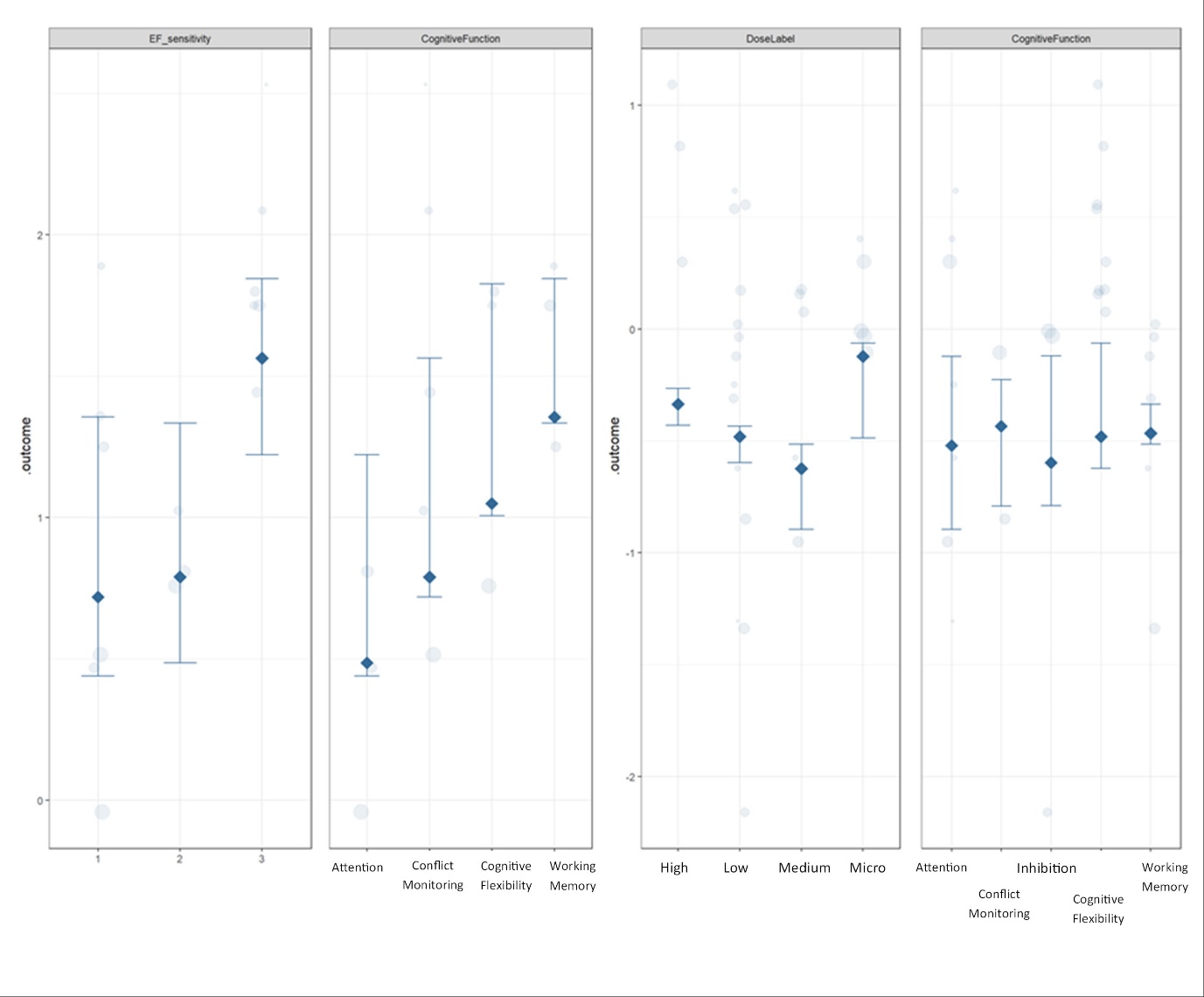


*Note.* Moderated partial dependence plots depicting the effects of psilocybin on RT and ACC. The left panel consists of two plots for RT: the first plot shows that lower levels of Executive Function sensitivity (higher values) are associated with longer RTs, while the second plot illustrates that tasks involving multiple executive functions and working memory lead to higher RTs compared to attention and conflict monitoring tasks. The right panel consists of two plots for ACC: the first plot indicates that micro doses of psilocybin are associated with higher ACC, whereas medium doses result in more negative outcomes. The second plot shows that the effect on working memory is the most precise.


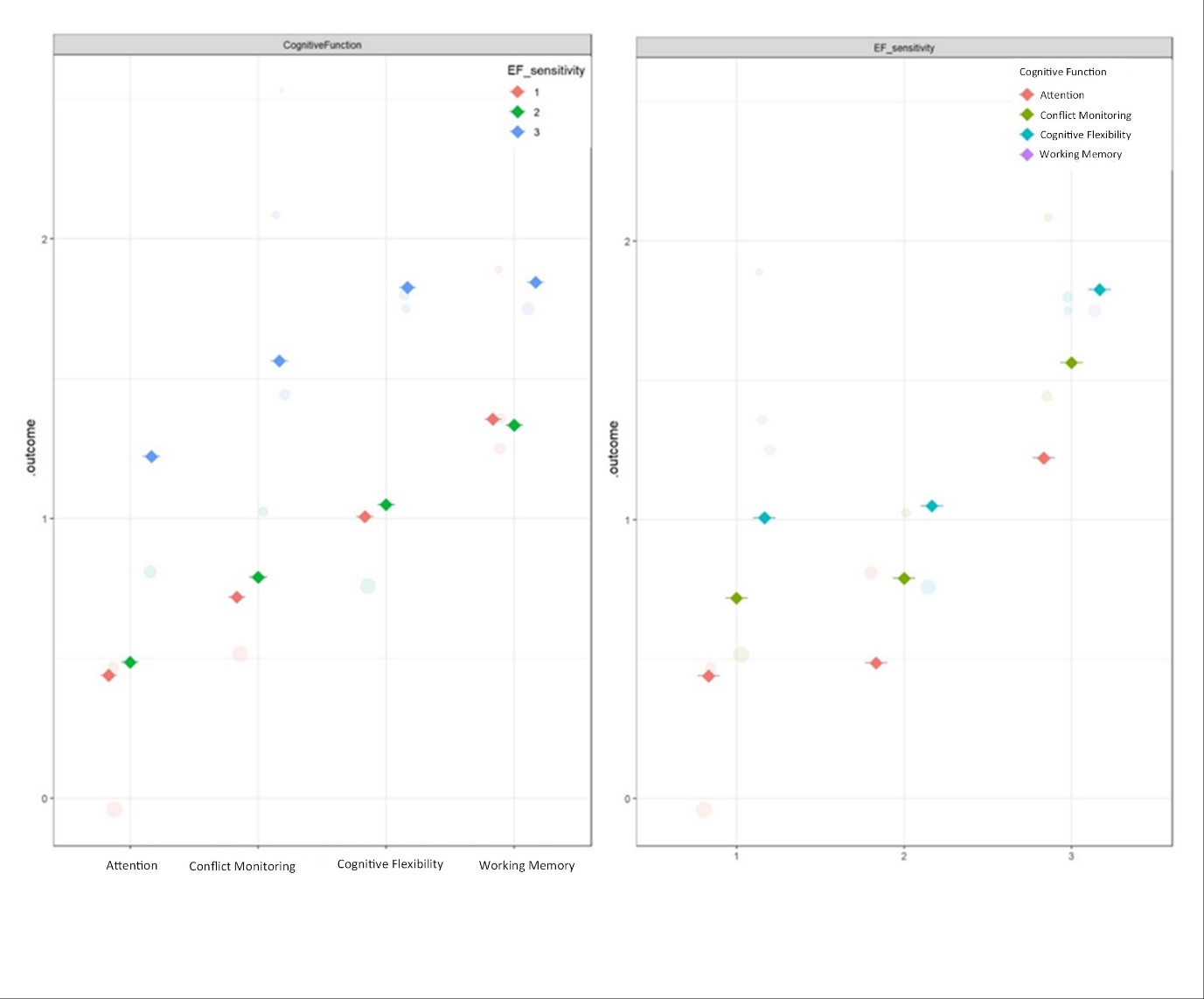
**Figure S4: Moderated Partial Dependence Plots Reaction Time**

*Note.* Moderated partial dependence plots depicting the influence of Executive Function (EF) sensitivity levels and different cognitive functions on RTs under the effect of psilocybin. The left panel shows the outcome (RT) by cognitive function, categorized by EF sensitivity levels (1, 2, 3; higher means less sensitive). The right panel displays the outcome (RT effect size) by EF sensitivity, categorized by cognitive functions (Attention, Conflict monitoring, Multiple Executive Functions, Working Memory).

**Figure S5: Moderated Partial Dependence Plots Accuracy**
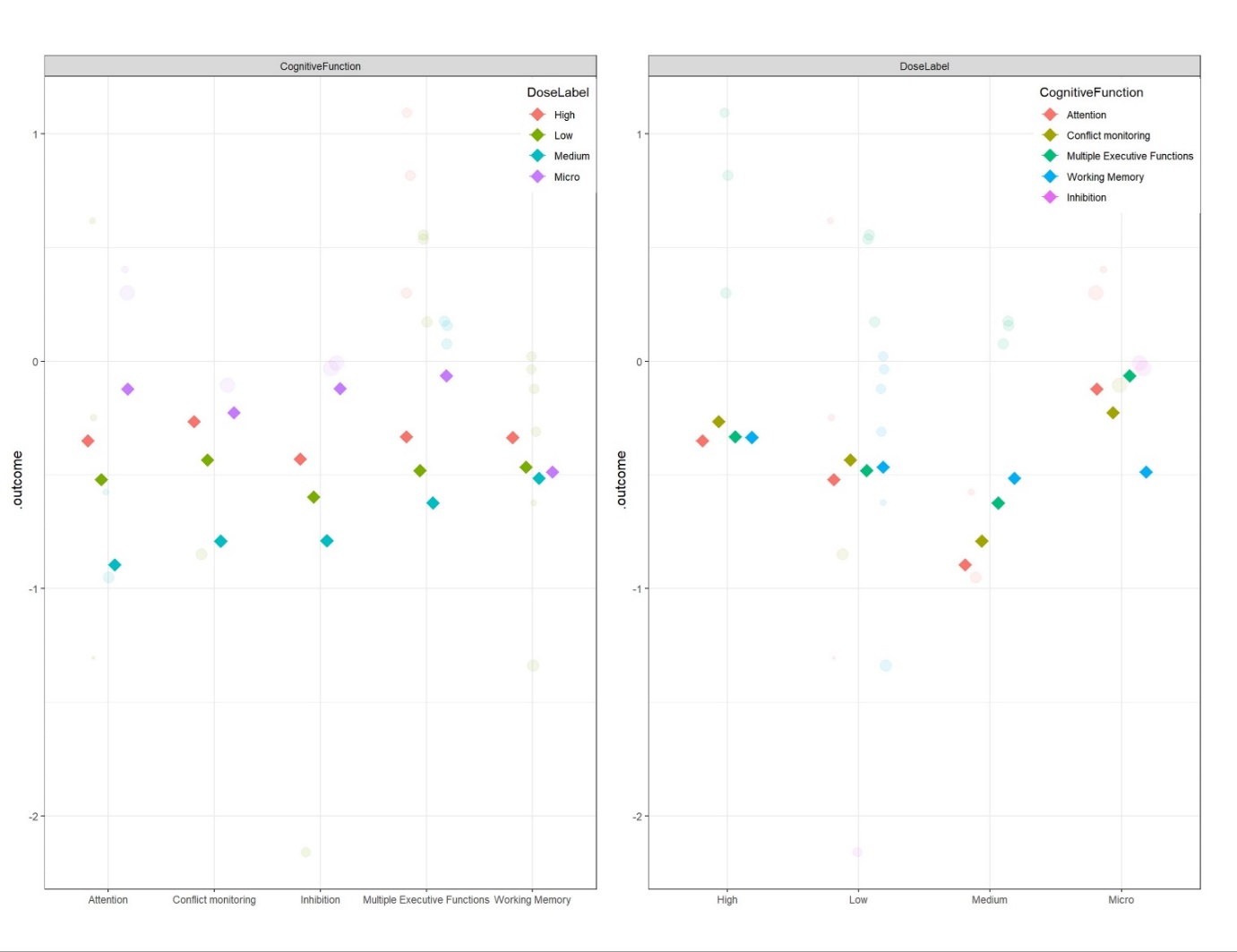


*Note.* Moderated partial dependence plots depicting the influence of dosage and different cognitive function categories on ACC under the effect of psilocybin. The left panel shows the outcome (ACC effect size) by cognitive function, categorized by dosage (micro, low, medium, high ). The right panel displays the outcome (ACC effect size) by dosage, categorized by cognitive functions (Attention, Conflict monitoring, Multiple Executive Functions, Working Memory, Inhibition).

**Figure S6: P and Z-Curve Analysis Reaction Time**
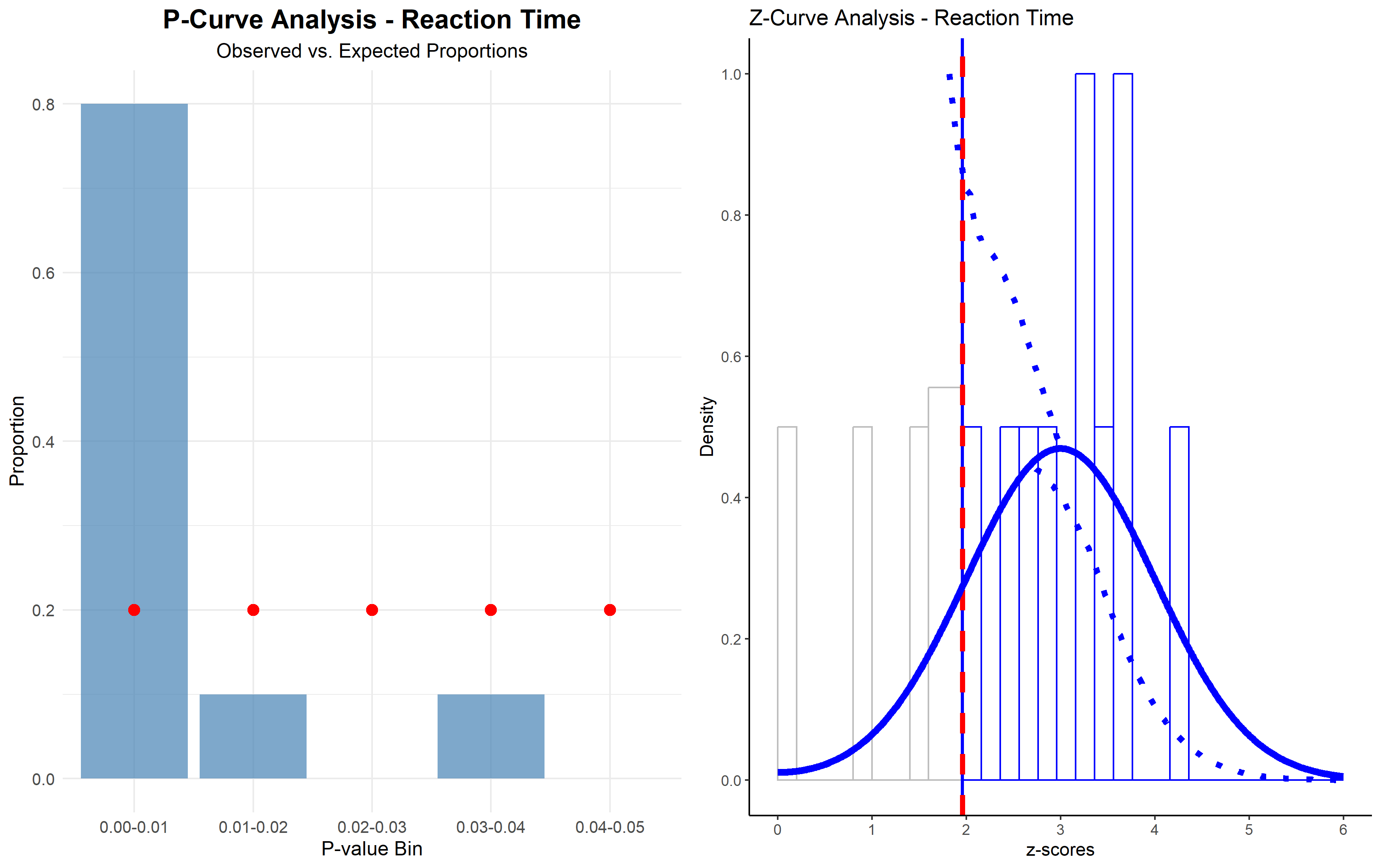


*Note.* P-curve and Z-curve analyses for RT data. The left panel shows the P-curve analysis, depicting the distribution of observed p-values (blue bars) against the expected uniform distribution (red dots) under the null hypothesis. The right panel presents the Z-curve analysis, illustrating the distribution of observed z-scores (blue line) and the estimated true power distribution (dotted blue line). The vertical red line represents the significance threshold (z = 1.96, p = .05). Both analyses suggest a right-skewed distribution of p-values and z-scores, indicating evidential value and potential true effects in the RT data.

**Figure S7: P-Curve Analysis Accuracy**


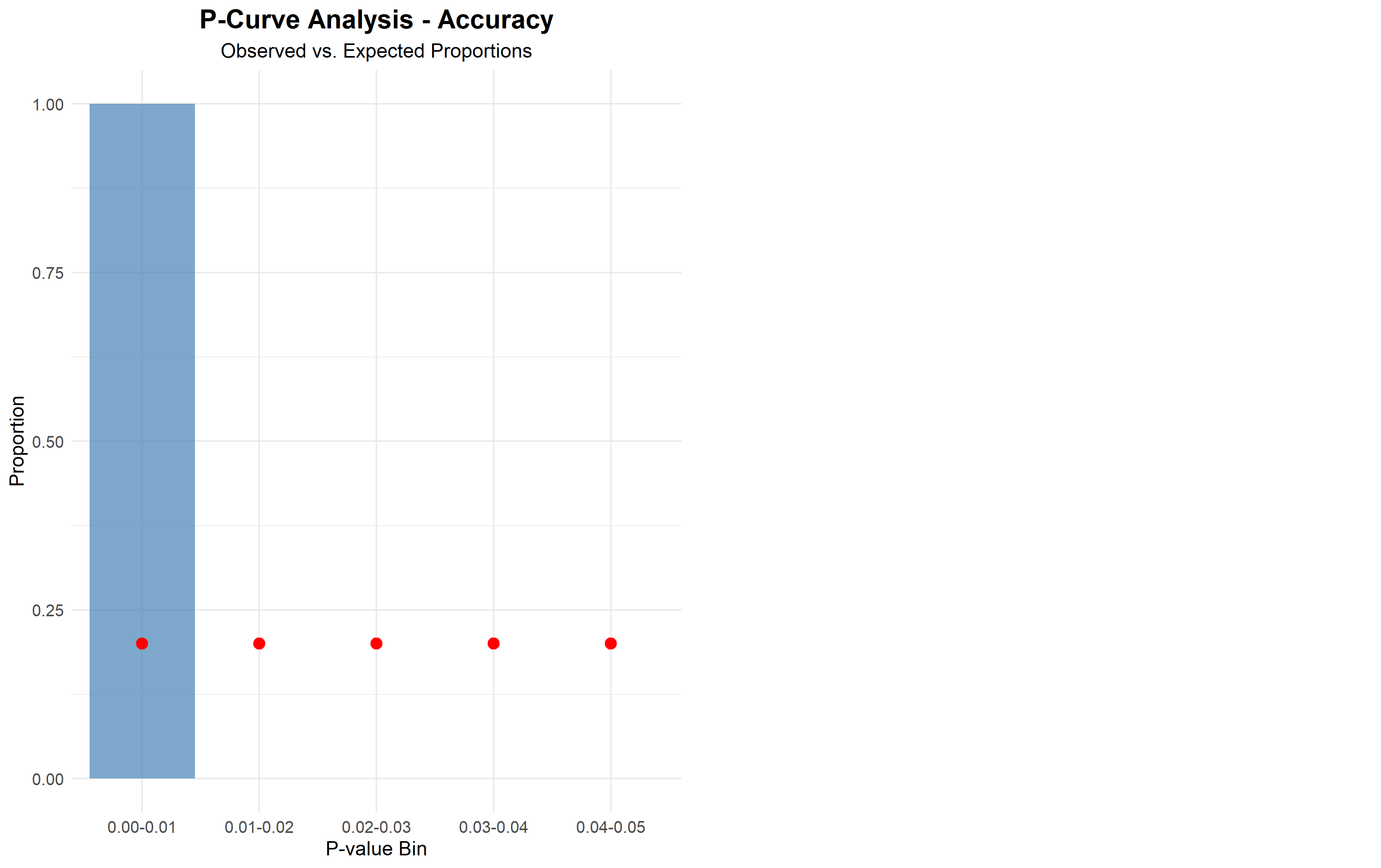


*Note.* P-curve analysis for ACC data. The graph shows the distribution of observed p-values (blue bars) compared to the expected uniform distribution (red dots) under the null hypothesis. Only the 0.00-0.01 bin contains significant p-values, with all other bins empty. This pattern, based on just two significant results, is insufficient for a robust z-curve analysis. The limited data precludes strong conclusions about evidential value or replicability for ACC effects.

**References**

Bartoš, F., & Schimmack, U. (2022). Z-curve 2.0: Estimating Replication Rates and Discovery Rates. *Meta-psychology*, *6*. https://doi.org/10.15626/mp.2021.2720

Simonsohn, U., Nelson, L. D., & Simmons, J. P. (2013, April 24). *P-Curve: A Key to the File Drawer*. https://papers.ssrn.com/sol3/papers.cfm?abstract_id=2256237

Strobl, C., Malley, J. D., & Tutz, G. (2009). An introduction to recursive partitioning: Rationale, application, and characteristics of classification and regression trees, bagging, and random forests. *Psychological Methods*, *14*(4), 323–348. https://doi.org/10.1037/a0016973

Van Lissa, C. J. (2017). MetaForest: Exploring heterogeneity in meta-analysis using random forests. *Open Science Framework*. https://doi.org/10.31234/osf.io/myg6
